# Slip-Spring and Kink Dynamics Models for Fast Extensional Flow of Entangled Polymeric Fluids

**DOI:** 10.3390/polym11030465

**Published:** 2019-03-11

**Authors:** Soroush Moghadam, Indranil Saha Dalal, Ronald G. Larson

**Affiliations:** 1 Department of Mechanical Engineering, University of Michigan, Ann Arbor, MI 48109, USA; soroushm@umich.edu; 2 Department of Chemical Engineering, Indian Institute of Technology Kanpur, Kanpur 208016, India; indrasd@iitk.ac.in; 3 Department of Chemical Engineering, University of Michigan, Ann Arbor, MI 48109, USA

**Keywords:** polymer rheology, extensional flows, slip-link simulations, kink dynamics, coil-stretch transition

## Abstract

We combine a slip-spring model with an ‘entangled kink dynamics’ (EKD) model for strong uniaxial extensional flows (with Rouse Weissenberg number $Wi_{R}\gg 1$) of long ($M_{w}>1\;\mathrm{Mkg}/\mathrm{mol}$ for polystyrene) entangled polymers in solutions and melts. The slip-spring model captures the dynamics up to the formation of a ‘kinked’ or folded state, while the kink dynamics simulation tracks the dynamics from that point forward to complete extension. We show that a single-chain slip-spring model using affine motion of the slip-spring anchor points produces unrealistically high tension near the center of the chain once the Hencky strain exceeds around unity or so, exceeding the maximum tension that a chain entangled with a second chain is able to support. This unrealistic tension is alleviated by pairing the slip links on one chain with those on a second chain, and allowing some of the large tension on one of the two to be transferred to the second chain, producing non-affine motion of each. This explicit pairing of entanglements mimics the entanglement pairing also used in the EKD model, and allows the slip spring simulations to be carried out to strains high enough for the EKD model to become valid. We show that results nearly equivalent to those from paired chains are obtained in a single-chain slip-spring simulation by simply specifying that the tension in a slip spring cannot exceed the theoretical maximum value of $\zeta^{\prime }\dot{\epsilon }L^{2}/8$ where $\zeta^{\prime }$, $\dot{\epsilon }$ and L are the friction per unit length, strain rate and contour length of the chain, respectively. The effects of constraint release (CR) and regeneration of entanglements is also studied and found to have little effect on the chain statistics up to the formation of the kinked state. The resulting hybrid model provides a fast, simple, simulation method to study the response of high molecular weight ($M_{w}>1\;\mathrm{Mkg}/\mathrm{mol}$) polymers in fast flows ($Wi_{R}\gg 1$), where conventional simulation techniques are less applicable due to computational cost.

## 1. Introduction

The ability to predict the flow behavior of polymeric fluids in strong flows has been highly sought over the past decades because of its relevance to applications in consumer products, pharmaceutical industries, electronics, etc. The molecular theory of Doi and Edwards [1,2] which is based on the “reptation” concept of De Gennes [3] has proved successful in describing the linear properties of entangled linear chains. The basic theory assumes that the polymer chain undergoes a one-dimensional diffusion inside a ‘tube’, where the tube is the mean-field approximation of the influence of the surrounding mesh of entanglements. During the past few decades, versions of tube theory have been proposed to include several relaxation mechanisms relevant to equilibrium and weak flows [4]. In general, a polymer chain in an entangled network relaxes through (1) reptation, (2) contour length fluctuations, and (3) constraint release; where the second and third mechanisms are related to the flexibility of the chain and relaxation of the surrounding network, respectively. A quantitative model combining these three relaxation physics was presented by Likhtman and McLeish [5] and its predictions were found to be in good agreement with the linear rheology of entangled monodisperse linear polystyrene (PS) and polybutadiene (PB) samples. In the nonlinear regime, when a strong flow is applied, other mechanisms need to be incorporated into the theory. The first of these is ‘segmental stretch’, in which the chain inside the tube stretches out due to the frictional force imposed by the surrounding matrix. This concept, introduced by Marrucci and Grizzuti [6] and further examined by Pearson et al. [7] for startup of simple shear flow, results in the Doi-Edwards-Marrucci-Grizzuti or ‘DEMG’ model [8,9]. The second mechanism, introduced by Marrucci [10], is ‘convective constraint release’ (CCR) whereby a fast flow sweeps away the spatial entanglement constraints around a sample chain, providing additional freedom for the chain to relax. Inclusion of CCR into the tube theory has resulted in different versions of the tube model for fast flows such as the Mead-Larson-Doi (MLD), double-convection-reptation with chain stretch (DCS-CS), GLaMM, “Rolie-poly” and Marrucci-Ianniruberto (M&I) models [11,12,13,14,15]. These models result in reasonably good agreement with experimental data in shear; however, poorer agreement is attained with experiments in transient fast extensional flows, where the polymer chain is highly stretched and aligned in the flow direction [16,17]. Although in some cases the disagreement is due to neglecting finite extensibility of the chain [13], it seems that there is a general disagreement for high molecular weight chains in fast flows, where the tube model predicts a much smaller Hencky strain ($\epsilon =\dot{\epsilon }t$) to reach the steady-state extensional stress ($\epsilon_{\mathrm{model}}^{\mathrm{ss}}$) than is observed experimentally ($\epsilon_{\exp}^{\mathrm{ss}}$) [18]. The recent addition of ‘friction reduction’ to the tube model in extension [19,20,21,22] has resulted in better agreement between model predictions and experimental values of the steady-state extensional viscosity ($\eta_{e}^{\mathrm{ss}}$), but this does not address the faster attainment of steady-state stress in the model compared to experiments. The inaccuracy of tube-model predictions of stress in extensional flows has attracted increasing attention in recent years and new simulation techniques [23,24,25,26] have been developed to shed light on the underlying dynamics of entangled polymers in fast extensional flows.

With recent advances in computational power, analysis of entangled polymers at the molecular level has become possible with the goal of improving the tube theory. In general, simulating the response of an entangled polymer chain can be done using (1) multiple-chain and (2) single-chain techniques. In the former, a simulation box is generated and filled with chains, wherein the monomers on different chains interact with each other and chain connectivity is conserved. Exemplary of this approach is the work of Kremer and Grest [27] who, by visualizing the motion of a primitive chain, they showed the existence of a tube-like region confining the motion of that chain in a melt at equilibrium. Applying this method to strong elongational flows at sufficiently large strains to reach steady state has in the past been challenging, due to excessive elongation and thinning of the simulation box in uniaxial flows [28,29,30]. While this limitation has now been overcome by a new method of re-mapping the box, still, performing these detailed simulations for long chains ($M_{w}>1\mathrm{Mkg}/\mathrm{mol}$) under high, but not unrealistically high, extension rates ($\dot{\epsilon }>1/\tau_{R}$, $\tau_{R}$. being the Rouse relaxation time of the polymer chain) remains computationally time consuming [24]. Therefore, coarse-grained versions of multiple-chains simulations (CG-MCS) have been developed, in which the basic length unit is chosen to be the subchain between consecutive entanglements. This is the approach taken in some slip-link simulatn methods, in which entanglements between chains are replaced by slip-links through which the polymer slides longitudinally [31,32,33,34,35,36]. The recently developed ‘primitive chain network’ (PCN) method has shown good agreement with experimental data for high molecular weight polystyrenes in uniaxial extensional flow at moderate Rouse Weissenberg numbers ($Wi_{R}=\dot{\epsilon }\tau_{R}<4$) [23]. However, the application of this method to higher Rouse Weissenberg numbers ($Wi_{R}>4$) has not been tested [23]. Another limitation of the PCN technique is the coarse-graining of the chain at the entanglement level, which results in loss of conformational details at distances smaller than the entanglement spacing [32]. We showed previously that, at high strains, kink formation and the unraveling dynamics govern the stress evolution [18]. In this manuscript, we analyze the chain statistics at the kinked state in detail and will show that kinks can form between entangled regions along a chain. The number of these unentangled kinks below the entanglement length-scale directly affects the stress evolution at high strains. Thus, chain configuration at the sub-entanglement level, which is ignored by methods such as PCN, is important.

To limit the required computational power, single-chain techniques have been developed, where the effect of the entanglement network is replaced by slip-links, which confine the movement of the chain and require the chain to slide through them to relax. The concept of slip-links was first introduced by Doi and Edwards [2] and further improved by Schieber and co-workers [37,38,39] who added additional dynamics such as constraint release and nonlinear effects. Single-chain slip-spring models have also been developed in which the effect of neighboring chains is replaced by virtual springs (or slip-springs) along the backbone of a single chain that not only confine the motion of the chain, but also exert force on the chain [40,41]. Despite their simplicity compared to multi-chain simulations, single-chain results match very well with linear viscoelastic data and nonlinear data in shearing flow, and can provide an intermediate level of detail between multiple-chain simulations and the tube theory [33,41,42,43,44,45,46]. However, predictions of single-chain simulations have not been very successful for strong extensional flows at high strains [45,47]. Moreover, even for these single-chain models, the computational time becomes unaffordably large as the chains become longer ($N_{k}>1000,\;N_{k}$ being the total number of Kuhn segments in a polymer chain) [48]. Therefore, there is a need for new simulation methods that can correctly predict the late-stage evolution of long chains in strong extensional flows ($Wi_{R}\gg 1$).

We recently presented a new simulation technique for arbitrarily long polymer chains in entangled systems that we call the ‘entangled kink dynamics’ (EKD) method [18] that predicts the final unraveling of a chain after it has been collapsed into a quasi-1D folded, or ‘kinked’ state by fast extensional flow. We showed that EKD predictions match well with experimental data for high molecular weight polystyrene in fast extensional flows beyond a Hencky strain of around ϵ=3, which is required to attain the folded state. In the present paper, we give a fuller presentation of our EKD approach. A new slip-spring simulation technique is also developed wherein we mutually pair the motion of entanglements on two chains, mimicking what is done in the EKD model, but using only two chains. The results of the new slip-spring model are compared with our previously used slip-spring method [18] and we show that both methods give similar results and appear to work well up to the transition strain at which the folded state is achieved, and beyond which the EKD method provides realistic predictions. Using the new slip-spring model, we obtain data on entanglement density and length distribution of strands between kinks in strong uniaxial extensional flow of linear chains are presented. With the new data, we redo our kink dynamics simulations and compare the results with experimental data of Huang et al. [49]. Moreover, we also study here the effects of constraint release and entanglement regeneration on the transition strain of chain [18], number of kinks on the chain, and entanglement density of kinks at the kinked state. In the following, in Section 2 we introduce our kink dynamics approach in full detail as well as the equations of motion for both dilute and entangled chains. Using results from the EKD model, we then develop a coarse-grained version of Likhtman’s slip-spring model [41] applicable to longer chain lengths and strong extensional flows, and carry out simulations with it in Section 3.1, Section 3.2 and Section 3.3. Finally, with the length distribution of strands and fraction of entangled kinks obtained using our slip-spring model, we return in Section 3.4 to the EKD model and compare its predictions with experimental data. We summarize in Section 4. Our multi-scale modeling using both slip-spring and kink-dynamics methods not only provides insight into the dynamics of entangled polymer under strong flow, but also predicts stresses that are in good agreement with experimental data in the literature. Our modified slip-spring model should also, with modifications, be useful for predicting other flows, such as shear.

## 2. Model and Simulation Method

### 2.1. Kink Dynamics Algorithm

The concept of kink dynamics in dilute solutions was first introduced in 1990 by Larson [50] and independently by Hinch [51]. Based on simulation studies of Rallison and Hinch [52], they suggested that an isolated, dilute, chain forms a folded state at large strain rates in which the chain consists of locally fully stretched and aligned chain segments connected by ‘kinks’ or back-folds, and it takes several Hencky strains ($\epsilon =\dot{\epsilon }t$) for the chain to completely unravel in uniaxial extension. This prediction was confirmed by subsequent experimental studies of dilute DNA solutions. Recent simulation studies of well entangled polymers have established that these folded, or ‘kinked’, states also form in entangled polymers at high extensional strains [18,25,53,54]. In fast extensional flows, in the kinked state, the magnitudes of the drag force and chain tension greatly exceed the Brownian force. Thus, one can neglect the effect of Brownian motion and assume that unraveling from a kinked to a fully unraveled state is driven by the balance of drag and tension. Numbering the kinks sequentially along the chain from 1 to $N_{\mathrm{Kinks}}$ (with chain ends taken to be ‘kinks’), we let $x_{i}$ be the x coordinate of kink i, ($1<i<N_{\mathrm{Kinks}}$), where the x direction is the stretching direction of the extensional flow. Balancing drag and tension forces on a small segment with a length dξ that lies between positions x=ξ and x=ξ+dξ, Larson derived the governing equation for the tension at position x of a chain in its folded state as [50]

(1) $$f^{s}\left(x\right)=-\frac{1}{2}\zeta^{\prime }\dot{\epsilon }\left(x^{2}-x_{i-1}^{2}\right)+\zeta^{\prime }V_{i}\left(x-x_{i-1}\right)+f_{i-1}$$

Here, $\dot{\epsilon }$ is the extension rate, $\zeta^{\prime }$ is the friction coefficient per unit length of the chain and $V_{i}$ is the velocity of strand (i) which connects kinks (i) and (i−1), and $f_{i-1}$ is the force at kink (i−1). We showed earlier that this equation can be re-written for the forces at three consecutive kinks (i−1, i, i+1) as ($\Delta x_{i}=x_{i}-x_{i-1}$) [18]

(2) $$\left(\frac{1}{\Delta x_{i+1}}\right)f_{i+1}+\left(\frac{1}{\Delta x_{i}}-\frac{1}{\Delta x_{i+1}}\right)f_{i}-\left(\frac{1}{\Delta x_{i}}\right)f_{i-1}=-\frac{1}{2}\zeta^{\prime }\dot{\epsilon }\left(x_{i+1}+2x_{i}+x_{i-1}\right)+2\zeta^{\prime }U_{i}\;$$

where $U_{i}$ is the velocity of kink (i) and is equal to $U_{i}=\left(V_{i}+V_{i+1}\right)/2$ due to conservation of the overall length of the chain. To solve Equation (2), we need to know either the forces or velocities of the kinks. For a dilute solution, due to the absence of any neighboring chains entangling with our chain of interest, the forces at the kinks go to zero ($f_{i}=0,\;1<i<N_{\mathrm{Kinks}}$) and the evolution of the system can be found by integrating the kink velocities to obtain the positions of the kinks as a function of time. We call the kinks that are not entangled with any surrounding chains and thus satisfy $f_{i}=0$, the ‘free kinks’. On the other hand, for a fully entangled chain, if one can assume that the chain resides inside a tube of entanglements that deform affinely with the macroscopically applied flow, then the kinks move affinely and for a kinked chain in uniaxial elongational flow, this implies $U_{i}=\dot{\epsilon }x_{i}$. Knowing the kinks’ velocities, we can find the evolution of the kink position as a function of time and calculate the stress. For the stress, we use the one-dimensional form of general Kirkwood–Riseman formula [55]

(3) $$\sigma_{xx}=\nu \langle \sum_{i=1}^{N_{\mathrm{Kinks}}}\left|\int_{x_{i-1}}^{x_{i}}f^{s}\left(x\right)dx\right|\rangle \;$$

Where ν is the number of molecules per unit volume and 〈 .〉 stands for the ensemble average. A summary of the equations of motion under dilute and affine-deformation assumptions are given in Table 1.

For affine motion, the forces $f_{i}$ at the kinks are found by solving the following system of linear equations, derived by explicitly writing Equation (2) for all the kinks on a chain (except for first and last kink, which, being chain ends, are free kinks):

(4) $$A\prime \left[\begin{array}{c} f_{2}\\ f_{3}\\ \vdots \\ f_{N_{\mathrm{Kinks}}-2}\\ f_{N_{\mathrm{Kinks}}-1} \end{array}\right]=\zeta^{\prime }\dot{\epsilon }\left[\begin{array}{c} -\frac{1}{2}x_{3}+x_{2}-\frac{1}{2}x_{1}\\ -\frac{1}{2}x_{4}+x_{3}-\frac{1}{2}x_{2}\\ \vdots \\ -\frac{1}{2}x_{N_{\mathrm{Kinks}}-1}+x_{N_{\mathrm{Kinks}}-2}-\frac{1}{2}x_{N_{\mathrm{Kinks}}-3}\\ -\frac{1}{2}x_{N_{\mathrm{Kinks}}}+x_{N_{\mathrm{Kinks}}-1}-\frac{1}{2}x_{N_{\mathrm{Kinks}}-2} \end{array}\right]$$

where A′ is given as

$$\left[\begin{array}{ccccccccccc} \left(\frac{1}{\Delta x_{2}}-\frac{1}{\Delta x_{3}}\right) & \frac{1}{\Delta x_{3}} & 0 & 0 & 0 & . & . & . & . & . & .\\ \frac{-1}{\Delta x_{3}} & \left(\frac{1}{\Delta x_{3}}-\frac{1}{\Delta x_{4}}\right) & \frac{1}{\Delta x_{4}} & 0 & 0 & . & . & . & . & . & .\\ 0 & \frac{-1}{\Delta x_{4}} & \left(\frac{1}{\Delta x_{4}}-\frac{1}{\Delta x_{5}}\right) & \frac{1}{\Delta x_{5}} & 0 & . & . & . & . & . & .\\ . & . & . & . & . & . & . & . & . & . & .\\ . & . & . & . & . & . & 0 & \frac{-1}{\Delta x_{N_{\mathrm{Kinks}}-3}} & \left(\frac{1}{\Delta x_{N_{\mathrm{Kinks}}-3}}-\frac{1}{\Delta x_{N_{\mathrm{Kinks}}-2}}\right) & \frac{1}{\Delta x_{N_{\mathrm{Kinks}}-2}} & 0\\ . & . & . & . & . & . & 0 & 0 & \frac{-1}{\Delta x_{N_{\mathrm{Kinks}}-2}} & \left(\frac{1}{\Delta x_{N_{\mathrm{Kinks}}-2}}-\frac{1}{\Delta x_{N_{\mathrm{Kinks}}-1}}\right) & \frac{1}{\Delta x_{N_{\mathrm{Kinks}}-1}}\\ . & . & . & . & . & . & 0 & 0 & 0 & \frac{-1}{\Delta x_{N_{\mathrm{Kinks}}-1}} & \left(\frac{1}{\Delta x_{N_{\mathrm{Kinks}}-1}}-\frac{1}{\Delta x_{N_{\mathrm{Kinks}}}}\right) \end{array}\right]$$

**Figure 1:**
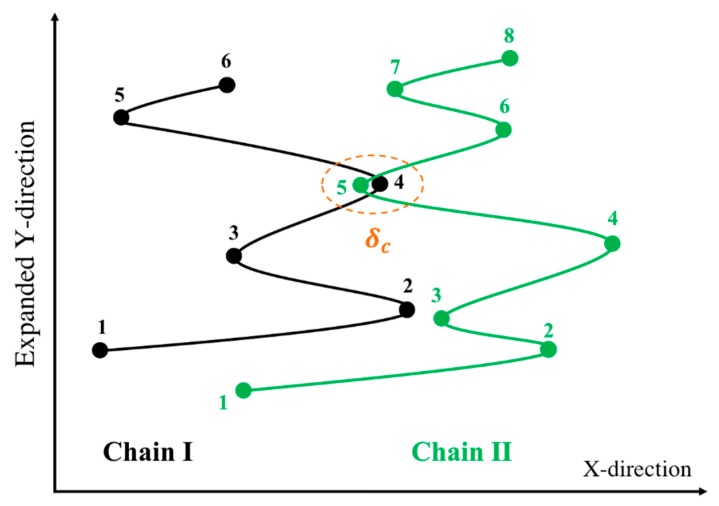
Schematic of an entangled kink between two chains in EKD model. The Y-direction above is arbitrarily expanded for clarity. Figure is adapted from [18] with permission, copyright American Chemical Society, 2019.

However, the affine motion of the entanglement network is a questionable assumption. In the tube theory, affine motion is a consequence of the mean-field nature of the tube as a representative of the confining effect of a large number of surrounding chains, large enough to represent the deformation of the continuum. Rubinstein and Panyukov, however, argued that the mesh of entanglements deforms non-affinely [56,57]. Multiple-chain PCN simulations [23] abandon the affine motion assumption and find the velocity of an entanglement point from a force balance between just two mutually entangled chains, rather than from the mean-field of many surrounding chains. These PCN simulations have yielded good predictions in both linear and nonlinear (shear and extensional flows) regimes [34], over the limited range of chain lengths, strain rates, and strains to which it has been applied. Following this idea, and presuming that entanglements are pairwise interactions of chains entangled mutually at the kinks, we developed our entangled kink dynamics algorithm (EKD) [18]. In this model, Equation (2) is solved for all entangled kinks without specifying either the force to be zero, as in dilute kink dynamics, or the velocity to be that of the continuum, as in the tube model. Instead, we require that both the force and velocity of the shared kink should be the same for the two chains. Figure 1 shows two chains, chains I and II, sharing an entangled kink and Equation (5) contains the four equations relating the force and velocity of the entangled kink on each chain as follows:

(5) $$\left\{ \begin{array}{c} \left[\left(\frac{1}{\Delta x_{5}^{I}}\right)f_{5}^{I}+\left(\frac{1}{\Delta x_{4}^{I}}-\frac{1}{\Delta x_{5}^{I}}\right)f_{4}^{I}-\left(\frac{1}{\Delta x_{4}^{I}}\right)f_{3}^{I}\right]-2\zeta^{\prime }U_{4}^{I}=-\frac{1}{2}\zeta^{\prime }\dot{\epsilon }\left(x_{5}^{I}+2x_{4}^{I}+x_{3}^{I}\right)\\ \left[\left(\frac{1}{\Delta x_{6}^{II}}\right)f_{6}^{II}+\left(\frac{1}{\Delta x_{5}^{II}}-\frac{1}{\Delta x_{6}^{II}}\right)f_{5}^{II}-\left(\frac{1}{\Delta x_{5}^{II}}\right)f_{4}^{II}\right]-2\zeta^{\prime }U_{5}^{II}=-\frac{1}{2}\zeta^{\prime }\dot{\epsilon }\left(x_{6}^{II}+2x_{5}^{II}+x_{4}^{II}\right)\\ U_{4}^{I}=U_{5}^{II}\\ f_{4}^{I}=f_{5}^{II} \end{array}\right.$$

**Figure 2:**
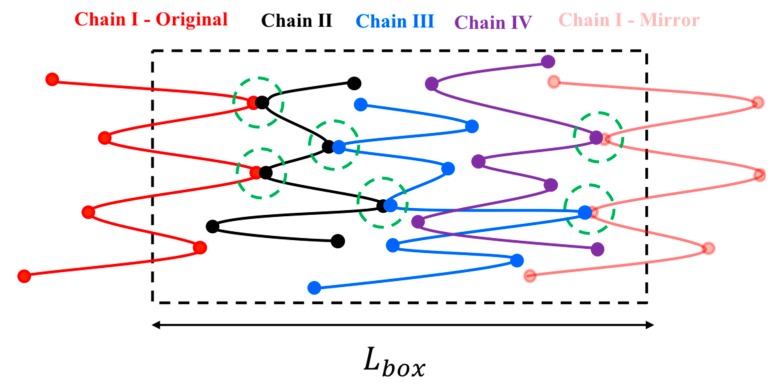
Periodic boundary condition applied at the simulation box limits. Mirror image of chain I (pale red) is entangled with chains III and IV shown in blue and purple, respectively. The original image of chain I is also entangled with chain II inside the simulation box. The schematic is adapted from our previous publication [18] with permission, copyright American Chemical Society, 2019.

Here, the subscript stands for the kink number and superscript for the chain index. The above four equations are used to solve for the four unknowns, $U_{4}^{I},U_{5}^{II},f_{4}^{I}$, and $f_{5}^{II}$. These four equations are coupled to equations for other kinks through the forces $f_{3}^{I}$ and $f_{6}^{II}$ that also appear in Equation (5). To simulate the response of an entangled system from the kinked state to the fully unraveled one, we generate a one-dimensional simulation box with length $L_{box}$ and randomly distribute $N_{\mathrm{chains}}$ in their kinked states within the simulation domain. Two kinks from two chains are considered entangled if (1) they have opposite polarity in the x-direction (the extensional axis) and (2) they are closer than a certain distance $\delta_{c}$. The force equations of the two kinks that are entangled at an entanglement point are coupled as specified by the system of Equation (5). Therefore, we have a system of $4\times N_{EP}$ equations where $N_{EP}$ is the number of entanglement points in the simulation box; each entanglement point is shared between two entangled kinks on different chains coupled at the entanglement point ($N_{EK}=2N_{EP}$, $N_{EK}$ being the total number of entangled kinks in the box). The pre-factor of 4 in ($4\times N_{EP}$) comes from the four equations we need to solve for each entanglement point, Equation (5). The initial entanglement fraction $\rho_{EK}^{0}$ is defined as the total number of entangled kinks ($N_{EK}$) divided by the total number of kinks ($N_{\mathrm{kinks}}$) and can be adjusted by changing $L_{box}$ and $\delta_{c}$. To obtain a statistically uniform distribution of entanglement points in the system, a periodic boundary condition (PBC) is applied at the simulation box limits, $x=-L_{box}/2$ and $x=L_{box}/2$. Note that without the PBC, all the kinks at the boundaries would be free. If a chain crosses the periodic boundary, its image is created on the other side of the box and the kinks of the image chain will be entangled with other chains, based on the polarity and adjacency criteria explained earlier; see Figure 2.

Special care needs to be taken in imposing velocities on mirror-image chains. In our system of equations, the velocity of a kink on a chain ($U_{i}^{o}$) and that on its mirror image ($U_{i}^{m}$) are related by

(6) $$U_{i}^{m}=U_{i}^{o}+\dot{\epsilon }L_{box}$$

Here, superscript (m) stands for ‘mirror’ and (o) for ‘original’. The difference between center of masses of the original and mirror-image chains is equal to the simulation box length ($L_{box}$). Constraint release is implemented self consistently by means of two mechanisms. First, when one of the chain ends passes through an entangled kink, the end kink of that chain is destroyed and the corresponding entanglement condition on the partner chain will disappear. Secondly, as two kinks at the ends of a shrinking strand meet, whether the kinks are entangled or free, they disappear. If one of the disappearing kinks is entangled with a partner chain, the paired kink of the neighboring chain becomes a free kink with zero tension. In the simulation approach defined so far, we have not implemented re-entanglement, so the number of entangled points in the system is an always decreasing function of simulation time. We previously showed, however, that re-entanglement does not have much effect on the behavior of EKD model [18].

#### Kink Dynamics Predictions for Entanglement Force

In earlier work, we used kink dynamics simulations to determine the evolution of chain conformation and stress under three conditions: 1) All free kinks, representing a dilute solution; 2) affine motion of kinks, representing the assumption of the tube model; and 3) pair-wise coupling of kinks. The first two approaches only require simulation of a single chain at a time, with averaging over an ensemble of such chains, while the third method requires simulation of a set of interacting chains using entangled kink dynamics (EKD). The following conclusions were obtained [18]:

1. Under affine motion, immediately after formation of the locally fully extended kinked state, the stress reaches at its final value which does not change during the unraveling of the kinked state to the fully extended state.
2. The chain end-to-end distance evolves very similarly under dilute and entangled kink conditions for a specific chain length.
3. Using the EKD model, when $\rho_{EK}^{0}=0.8$ (a highly entangled system), the tension along the chain at the start of the folded state is almost flat and as the chain unravels, it becomes quadratic after a few Hencky strain units, depending on the length of the chain.

**Figure 3:**
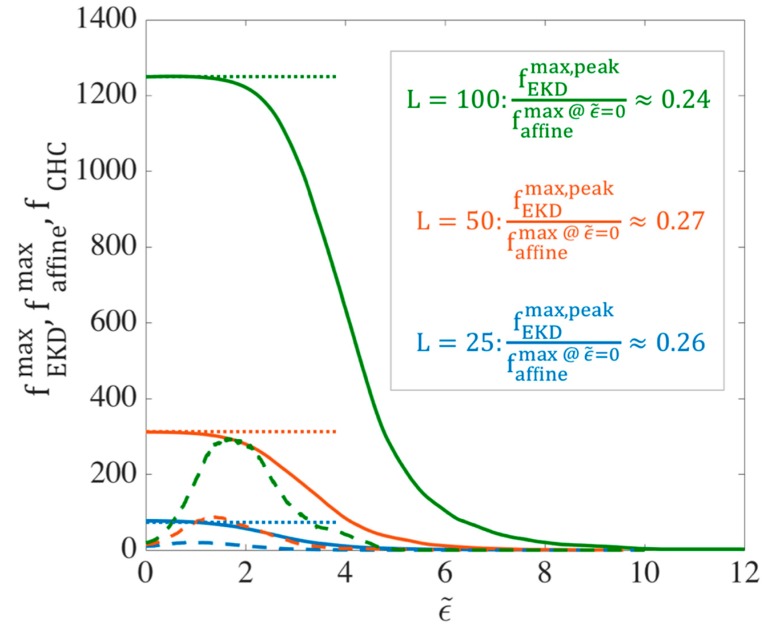
Evolution of the ensemble-averaged maximum kink force ($f_{kink}^{max}$) for different chain lengths L=25 (blue), L=50 (orange), and L=100 (green) at asymptotically high strain rates. For each length, the ensemble averaged force is shown for both affine motion of kinks (solid lines) and EKD simulations with $\rho_{EK}^{0}=0.8$ (dashed lines). The horizontal dotted lines give the maximum possible force, $f_{CHC}$ at an entangled kink for each chain length, obtained from Equation (7) as discussed in the text. The units of the force are set by the parameter values $\zeta^{\prime }=\dot{\epsilon }=1$; i.e., the dimensional force is obtained by multiplying the plotted force by $\zeta^{\prime }\dot{\epsilon }$ times the square of the unit of length.

In this section, we determine the chain tension predicted by the EKD model, in particular the maximum tension that an entangled kink can experience. For all simulation results shown in this section, we take $\nu =\zeta^{\prime }=\dot{\epsilon }=1$. Specifically, we plot the maximum force generated at an entangled kink ($f_{\mathrm{kink}}^{\max}$) under affine and EKD assumptions. This calculation gives us the maximum entanglement force that is generated by the drag on the two entangling kinked chains in the kinked state. As we will discuss in Section 2.2, this maximum force at an entanglement is required for the slip-spring simulations. To calculate the average maximum entanglement force, for each chain in our ensemble at a given strain, we first find the maximum force at any kink on that chain. Then, we average this maximum force over all chains in the ensemble and call this the average maximum kink force $f_{\mathrm{kink}}^{\max}$. Figure 3 shows the evolution of $f_{\mathrm{kink}}^{\max}$ when the chains deform affinely (solid lines) and using the EKD technique (dashed lines) for three chain lengths, L=25, 50 and 100. Here and elsewhere, $\tilde{\epsilon }$ is the strain starting from the kinked state and ϵ denotes the total strain, starting from the equilibrium. $\tilde{\epsilon }$ and ϵ are connected by $\epsilon_{T}$, which is the strain at which folded state of the chain is achieved, so we have $\tilde{\epsilon }=\epsilon -\epsilon_{T}$. We previously estimated from slip-spring simulations $\epsilon_{T}\sim 3$ [18], and we further investigate the appropriate value in this manuscript. For affine-motion simulations, we solve the system of Equations (4) at each strain ($\tilde{\epsilon }$) and find the maximum of the kink forces, $f_{i}$’s, for each chain. In EKD simulations, the system of Equations (5) is solved for all entangled kinks and the maximum value of the $f_{i}$’s for each chain is found. For both affine-motion and EKD simulations, we averaged these maximum values over multiple chains ($N_{\mathrm{chains}}=100$).

**Figure 4:**
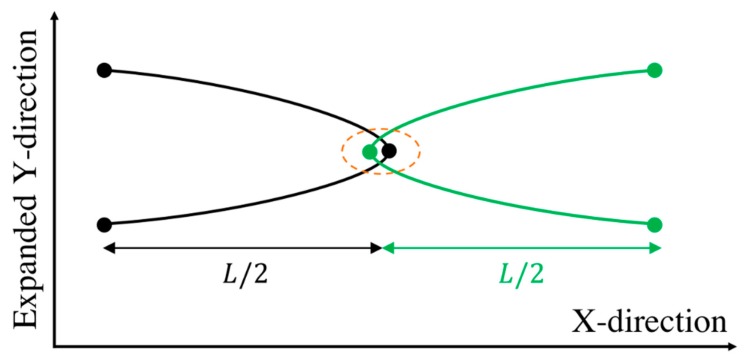
The ‘center-hooked’ configuration between two chains entangled at the center of each chain. The Y-direction is arbitrarily expanded for clarity.

Taking an entanglement as a pairwise interaction between two chains, the maximum possible force at an entangled kink between two chains is achieved when the two chains are symmetrically entangled at the center of each chain; see Figure 4. We call this the ‘center hooked conformation’ (CHC). Solving the system of Equations (5) for the center hooked conformation, the force at the entangled kink in the middle, which is the maximum possible force, is found to be:

(7) $$f_{\mathrm{CHC}}=\frac{1}{8}\zeta^{\prime }\dot{\epsilon }L^{2}$$

**Figure 5:**
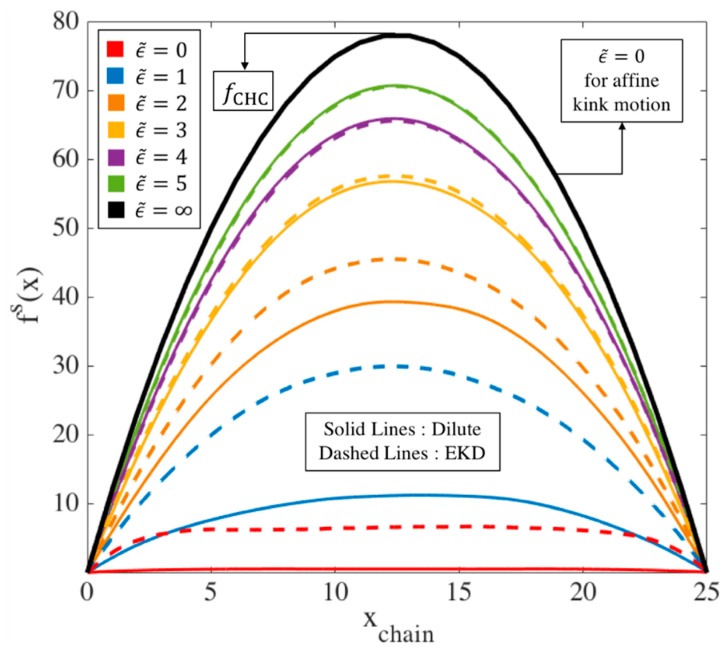
Evolution of tension distribution ($f^{s}\left(x\right)$) along an L=25 chain under affine motion, and for dilute and entangled chains, the latter simulated by the EKD method and averaged over an ensemble of 150 chains. The arrow at the peak of black curve shows the maximum tension value a chain of length L=25 will achieve when fully unraveled for a dilute and an EKD chain, which is attained immediately in the kinked state under affine motion of kinks. The peak value of this maximum tension is given by Equation (7).

Values of $f_{\mathrm{CHC}}$ for three chain lengths are plotted as dotted horizontal lines in Figure 3. Figure 3 reveals that for affine deformation, $f_{\mathrm{affine}}^{\max}$ initially takes this highest achievable value, $f_{\mathrm{CHC}}$, which is at the center of the chain with force away from the center distributed along the quadratic curve shown by black line in Figure 5. As the chain unravels under affine motion, kinks near the chain center move away from each other and the strand length between them increases. With fewer kinks and fewer forces, the maximum of these is less likely to be near the peak of the black curve and more likely to be off to the side, and lower in value, producing the decrease in maximum force $f_{\mathrm{affine}}^{\max}$ shown by the solid lines in Figure 3.

On the other hand, for the same chain length, with 80% initial entangled kinks in an EKD simulation, the evolution of the maximum kink force is drastically different as shown in Figure 3 by the dashed lines. First, the peak occurs at a finite strain (~1−2 Hencky strains) rather than instantly after startup of the simulation and also the peak tension is much smaller than for affine motion. In EKD simulations, the tension distribution along the whole chain (not just at the kinks) is initially nearly uniform, except at the chain ends, and then eventually evolves into a quadratic dependence, which takes almost 6 Hencky strain units to occur for the chain length considered. The tension distribution of a dilute chain, also plotted in Figure 5, is initially lower than that of the entangled chain, but merges with it at $\tilde{\epsilon }=4$. This is because no re-entanglement is implemented, so that after annihilation of entangled kinks due to unraveling, the entangled chain becomes dilute and loses all its entanglements by $\tilde{\epsilon }\sim 4$.

We observe in Figure 3 that the peak value of $f_{\mathrm{EKD}}^{\max}$ in the EKD simulations for each chain length is much smaller than the initial value obtained in affine motion, by roughly a factor of four. Thus,

(8) $$f_{\mathrm{EKD}}^{\max,\mathrm{peak}}≅0.25\;f_{\mathrm{affine}}^{\max,\;@\;\tilde{\epsilon }=0}≅0.25\;f_{CHC}=\frac{1}{32}\zeta^{\prime }\dot{\epsilon }L^{2}\;$$

The next step is to apply the kink dynamics analysis to experimental polymer solutions, e.g., the polystyrene samples of Hunag et al. [49]. To do so, the following questions need to be answered:

1. What is the transition strain ($\epsilon_{T}$) beyond which the EKD results become valid?
2. What is the number of kinks for a chain of arbitrary chain length at the transition strain?
3. What is the ratio of the number of entangled to free kinks in an entangled sample?
4. What is the distribution of strand lengths between the kinks?

To answer above questions, we need a simulation technique that can track the evolution of the chain conformation from equilibrium at least to the kinked state. For this purpose, we perform Brownian dynamics simulations of entangled chains using the single-chain slip-link model of Likhtman [41]. In this model, entanglement forces on a chain are captured by slip springs that are anchored to the continuum. The anchor points are held fixed in position for simulations of linear viscoelasticity and move affinely in the case of imposed flow. This model has been shown to predict well the linear rheology and stresses in shear flow of entangled polymers [33,43,58], where the forces generated by entangled chains are small or modest. However, as emphasized above, in uniaxial extensional flow, the tension in the chain can become large enough to cause large deviations from affine motion of entanglement points. Thus, after introducing the slip-spring model, will address this limitation of the original slip-spring model, by either pairing slip springs on one chain with those on another, to allow non-affine motion of the anchor point based on a simple force re-distribution condition, or by simply imposing a maximum-force constraint on the slip spring. We will show that both methods, though rather ad hoc, give similar results up to the transition to the kinked state, and fix the severe problems that the model otherwise encounters in extensional flow.

### 2.2. Slip-Spring Simulations

Kink dynamics simulations are only valid after the chain has reached a folded one-dimensional conformation with strands between folds almost fully extended. Larson used an approximation to determine the mean distance between two folds, $\Delta x=b_{k}{\left(N_{k}^{2}/2\pi^{2}\tau_{R}\dot{\epsilon }\right)}^{1/3}$ and average number of kinks $N_{\mathrm{kinks}}=L/\Delta x={\left(2\pi^{2}N_{k}\tau_{R}\dot{\epsilon }\right)}^{1/3}$ at the folded state for dilute solutions, where $N_{k}$ and $b_{k}$ are the total number of Kuhn steps and Kuhn step length in a polymer chain, respectively [50]. Here, going beyond a simple scaling analysis, we perform Brownian dynamics (BD) simulations of an entangled polymer using the slip-link model of Likhtman [41].

**Figure 6:**
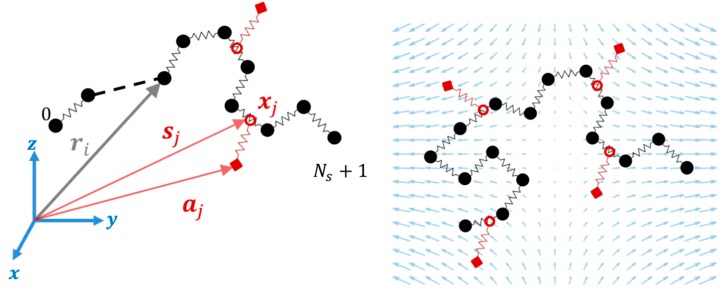
Schematic of slip-spring simulation in an extensional flow. Position vector of a bead ($r_{i}$), anchoring point of a slip-spring ($a_{j}$), position of a slip-link on the chain ($s_{j}$), and a dummy variable ($x_{j}$) of a slip-spring along a chain are shown on the left. On the right, a uniaxial extensional flow is applied to this single-chain model of an entangled polymer, where each slip-spring anchoring point (red squares) moves affinely with the flow until the force generated in the virtual spring (red) reaches a maximum value.

In the single chain slip-link model, a freely-jointed chain, with $N_{s}$ springs between $N_{s}+1$ beads, is generated in three dimensions. Each spring in the chain represents $N_{k,s}$ Kuhn segments with Kuhn length $b_{k}$ resulting in a total of $N_{k}=N_{k,s}N_{s}\;$ Kuhn steps and a contour length of $L=N_{k,s}b_{k}$ for the chain. Each bead is a center of friction with a friction coefficient $\zeta_{b}$. Slip-springs add potentials generated at random positions around the polymer chain. Each slip-spring is extended between ${\vec{a}}_{j}$ (its anchoring point) and ${\vec{s}}_{j}$ (location of slip-link) as shown in Figure 6 [41]. There are $N_{k,ss}$ Kuhn steps in each slip-spring with the same Kuhn length $b_{k}$ as in the main chain, so that the maximum length of a slip-spring is $L_{ss}^{\max}=N_{k,ss}b_{k}$. The initial number of slip-springs ($Z_{0}$) is determined by the number of beads between slip-springs ($N_{e}$), so that $Z_{0}=N_{s}/N_{e}$. To find the location (${\vec{s}}_{j}$) of a slip-link j (where *j* runs from 1 to the total number of slip links Z) on the chain, Likhtman used a continuous dummy variable ($x_{j}$), which can take any real value between 0 and $N_{s}$, so that $0<x_{j}<N_{s}$. The dummy variable determines the relative position of the slip-link on a main chain spring with respect to the neighboring beads connected by that spring. Positions of beads on the main chain evolve due to drag, elastic forces exerted by connecting springs, Brownian and slip-spring forces, and the slip-link moves along the chain under the effect of slip-spring and Brownian forces [18]. Slip-links move along the chain with a friction coefficient ($\zeta_{s}$) different from that of the beads ($\zeta_{b}$). Likhtman studied the effect of $\zeta_{s}$ on the viscosity, and found that a value of $\zeta_{s}=0.1$ (in the dimensionless units below) gives reasonable results; this value has been used in other slip-spring studies as well [33,41,43], and we will use this value here. Since we want to apply strong extensional flows to the bead-spring system, we use the Cohen–Pade finitely extensible force law for both the springs on the main chain and the slip-springs [18].

Simulations are performed in a dimensionless form by reducing the dimensions of force, time and length by $k_{B}T/b_{k}$, $\zeta_{b}b_{k}^{2}/k_{B}T$, and $b_{k}$ respectively, which differs from the dimensionless variables used in the kink dynamics simulations. An explicit Eulerian forward time stepping is used to integrate the equations. The Rouse time of the coarse-grained chain is given by

(9) $$\tau_{R}=\frac{\zeta_{b}{\left(N_{s}+1\right)}^{2}N_{k,s}b_{k}^{2}}{6\pi^{2}k_{B}T}\Rightarrow \tau_{R}^{*}=\frac{{\left(N_{s}+1\right)}^{2}N_{k,s}}{6\pi^{2}}\Rightarrow Wi_{R}={\dot{\epsilon }}^{*}\tau_{R}^{*}\;$$

where the superscript (*) denotes the dimensionless value and $Wi_{R}$ is the Rouse-Weissenberg number of the chain. In the following, we report dimensionless values of the simulation parameters without the (*) for simplicity. Simulation inputs are given in Table 2.

Here we use $N_{e}=4\;$and $N_{k,ss}/N_{k,s}=0.5\;$as suggested by Likhtman. However, our simulations differ from those of Likhtman in that we coarse grain a bit further and use $N_{k,s}=5$ Kuhn steps for the springs on the main chain instead of $N_{k,s}=1$ used by Likhtman and coworkers. Using a larger number of Kuhn steps for the springs of the main chain, in comparison to what Likhtman used, make the chain more flexible and acts as a diluting effect, since the number of Kuhn segments between two consecutive entanglements increases to 20 in our simulations compared to Likhtman’s four Kuhn segments. Therefore, the entangled chains in our slip-spring simulations are about 20% more dilute than a melt, which justifies the use of experimental data of entangled solutions, with concentration similar to this, to validate the model. Given $N_{k,s}=5$, we use $N_{k,ss}$ = 2.5 based on the given ratio $N_{k,ss}=0.5N_{k,s}$ suggested by Likhtman [41] and Sukumaran and Likhtman [59]. With this coarse-graining relative to the original model, the number of Kuhn steps in each slip-spring is half that of the springs in the main chain for our coarse-grained chain with $N_{e}=4$. The use of this somewhat more coarse-grained version of the model allows us to reach longer chain lengths and avoid overly small time steps in fast extensional flow.

**Figure 7:**
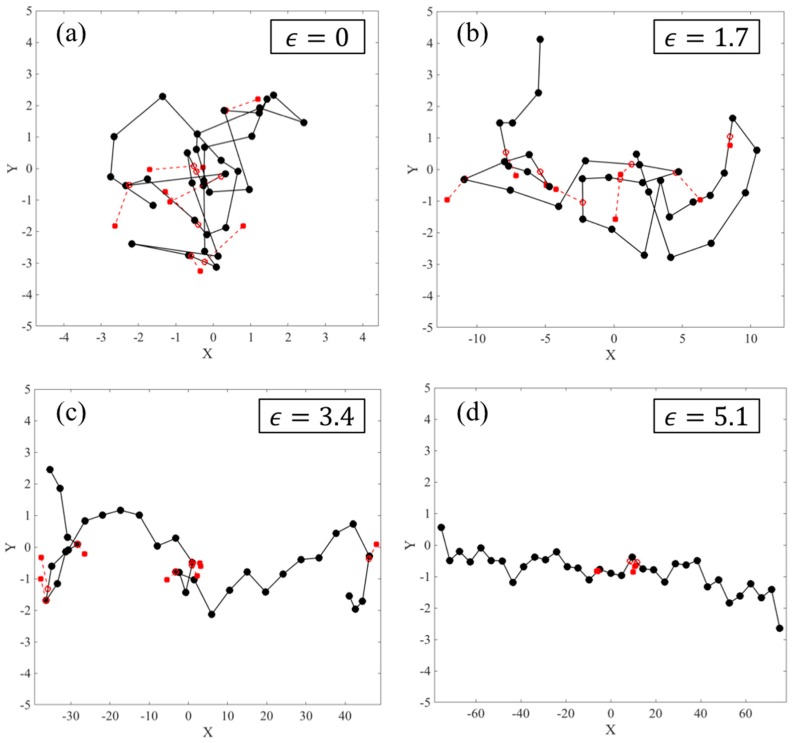
Evolution of a sample chain with $N_{s}=32$ ($Z_{0}=8$) at $Wi_{R}=16$, at Hencky strains of (**a**) ϵ=0, (**b**) ϵ=1.7, (**c**) ϵ=3.4, and (**d**) ϵ=5.1. Notice the x scale, but not the y scale, is increased in range at large strains. Main chain beads and springs are shown with black circles and lines, respectively, while slip-springs are shown in red, extending from their anchoring points marked with solid red circles to their positions on the chain, marked with open red circles. A maximum slip-spring force is used, and regeneration of slip-springs is turned off.

**Figure 8:**
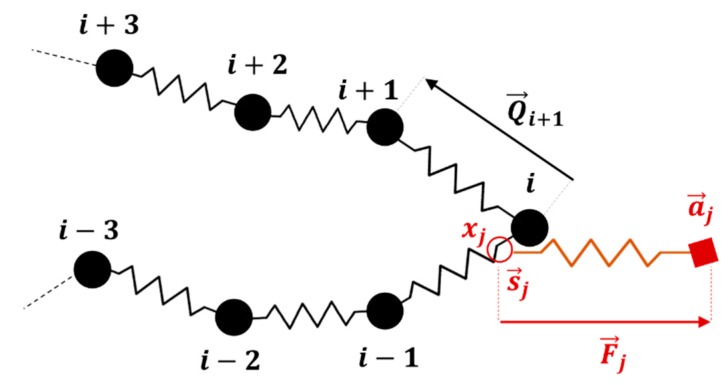
Trapping of slip-spring in the kinked region between three beads that define the folded area. The anchoring point ($a_{j}$) moves affinely with the flow, while the position of the slip-link on the chain, determined by Equations (8) and (10), oscillates between spring ${\vec{Q}}_{i}$ and ${\vec{Q}}_{i+1}$.

Before applying the extensional flow, all the chains in the ensemble are simulated in the absence of flow for a time $5\tau_{R}^{*}$ to relax the chain from its initially generated random conformation and ensure that the chain has reached equilibrium. During the equilibrium process only, we keep the number of slip-springs on the chain constant, by generating a new slip-spring at a random position along the chain whenever a slip-spring is released from the chain end. Therefore, during relaxation, the number of slip-springs is fixed at $Z_{0}$. To perform these equilibration simulations, the drag term on the right-hand side of Equation (3) of our previous publication [18] is simply set to zero. When the flow is applied, the anchoring points of the slip-springs (${\vec{a}}_{j}$) move affinely with the flow and the connecting vectors of the slip-springs increase in length gradually. While slip links on the chain are lost as they are convected off the chain, neither destruction of slip links by any constraint-release from the surrounding medium, nor regeneration of slip links are considered. Thus, the number of slip-springs on the chain (Z) starts from $Z_{0}$ and decreases to zero as the chain gradually achieves its fully unraveled state. As the chain orients in the flow direction and its springs stretch out, due to the imposed drag and slip-spring forces, the chain evolves into a folded state and kinks are formed. Figure 7 shows a sample chain of 32 springs evolving from the equilibrium state to a fully unraveled chain at $Wi_{R}=16$. After the kinks appear in the chain at strains close to 2.5, some of the slip-links move towards the kinks and make an entangled kink. At this point the slip-link cannot escape the kinked region until the kink disappears by chain unraveling. We call this situation ‘slip-link trapping’, since the slip-link is trapped between the three beads that form the fold, Figure 8, and cannot release itself unless the kink disappears or constraint release (which has not been implemented in this section) removes the trapped slip-link. As described below, trapping occurs because the flow affinely drives both the kink and the slip link in the same direction. Rewriting Equation (4) of [18] in its numerically integrated form, the position $x_{j}$ of slip-link (j), evolves as

(10) $$x_{j}^{t+\Delta t}=x_{j}^{t}+\frac{\Delta t}{\zeta_{s}}\;\left({\vec{F}}_{j,SS}.\left({\vec{r}}_{x_{j}+1}-{\vec{r}}_{x_{j}}\right)\right)+\Delta x_{B}$$

where ${\vec{F}}_{j,SS}$ shows the force in slip-spring (j). As the magnitude of the force in the slip-spring ($\left|{\vec{F}}_{j,SS}\right|$) increases, the second term on the right-hand-side of Equation (10) becomes dominant, and one can ignore the effect of Brownian motion $\Delta x_{B}$. As shown in Figure 8, when trapping occurs, the direction of the force ${\vec{F}}_{j,SS}$ stays constant, but as the slip-link travels between beads i−1, i and i+1, when it passes bead *i*, the sign of the position term (${\vec{r}}_{x_{j}+1}-{\vec{r}}_{x_{j}}$) in Equation (10) reverses. Thus, if $x_{j}$ increases during one time-step, it will decrease in the next, so that the position of slip-link (${\vec{s}}_{j}$) jumps from its location on the spring whose vector is ${\vec{Q}}_{i}={\vec{r}}_{i}-{\vec{r}}_{i-1}$ (see Figure 8), to a location on ${\vec{Q}}_{i+1}$, and so on. Therefore, one end of the slip-spring, ${\vec{s}}_{j}$, fluctuates in the kinked region, while the other end, ${\vec{a}}_{j}$, moves affinely with the flow, independent of the chain conformation.

This trapping dynamics in the kinked region results in a nearly complete extension of the slip-springs and consequently, high values of tension generated in the slip-springs, which exert non-physically high magnitudes of force on the main chain. The high slip-spring tension also causes slip-spring breakage when the spring stretches beyond its maximum extension within a single time state, even with very small Δt. Note that if the polymer chains were more highly resolved, say at the level of a Kuhn step, local Brownian motion would more readily allow beads on the chain to cross the slip link and trapping would then not occur. The chain would then slide through the slip link, which would keep the slip-spring force from exceeding the maximum chain tension for affine deformation, calculated in Equation (7). However, such a highly resolved chain would require much more computer time, and the advantage of the slip-spring algorithm over finer grained molecular dynamics simulations would largely be lost. Therefore, instead of a more highly resolved simulation, an upper limit of slip-spring force ($F_{\mathrm{ss}}^{\max}$) can be imposed to prevent the overextension of slip-springs and to keep the entanglement force within the physically meaningful range determined earlier by our kink dynamics analysis. For affine kink motion, the maximum entanglement force is given by $\zeta^{\prime }\dot{\epsilon }L^{2}/8$ (Equation (7)) and for EKD simulations, the actual average maximum force was found to be approximately four times lower, $\zeta^{\prime }\dot{\epsilon }L^{2}/32$. Since we initially impose affine deformation on the anchoring points of the slip-springs, we use the theoretical maximum force value $\zeta^{\prime }\dot{\epsilon }L^{2}/8$ rather than the average maximum actually attained $\zeta^{\prime }\dot{\epsilon }L^{2}/32$. Thus, from the kink dynamics analysis, we set a maximum entanglement force for the slip-springs that depends on the dimensionless contour length of the chain, $L=N_{k,s}N_{s}$, the strain rate $\dot{\epsilon }$ and the friction coefficient per unit length $\zeta^{\prime }$. The value of $\zeta^{\prime }$, the friction coefficient per chain length that can be calculated from the Rouse theory, and the corresponding dimensionless value ${\zeta^{\prime }}^{*}$, are given as [50]

(11) $$\left\{ \begin{array}{l} \zeta^{\prime }=\frac{6\pi^{2}kT\tau_{R}}{b_{k}^{3}N_{k}^{2}}=\frac{6\pi^{2}kT}{b_{k}^{3}N_{s}^{2}N_{k,s}^{2}}\tau_{R}\\ \;\tau_{R}=\frac{\zeta_{b}{\left(N_{s}+1\right)}^{2}N_{k,s}b_{k}^{2}}{6\pi^{2}kT} \end{array}\right.\Rightarrow \zeta^{\prime }=\frac{\zeta_{b}}{b_{k}}\frac{{\left(N_{s}+1\right)}^{2}}{N_{s}^{2}}\frac{1}{N_{k,s}}\to {\zeta^{\prime }}^{*}=\frac{{\left(N_{s}+1\right)}^{2}}{N_{s}^{2}}\frac{1}{N_{k,s}}$$

When the force in the slip-spring exceeds $F_{\mathrm{ss}}^{\max}=f_{\mathrm{affine}}^{\max}=\zeta^{\prime }\dot{\epsilon }L^{2}/8=6\pi^{2}Wi_{R}/8$, we therefore modify the position of the anchoring point by reducing the stretch of the connecting vector, without changing its direction, so that its force equals the maximum entanglement force. To do so, each time step the maximum force is exceeded, the anchoring point must be shifted backwards along the flow direction relative to the position the flow would otherwise take it, resulting in a non-affine motion of anchoring points at high strains where trapping tends to occur. If the force in the slip-spring is less than the maximum, the anchoring points move affinely. The expression $F_{\mathrm{ss}}^{\max}=6\pi^{2}Wi_{R}/8$ is obtained by combining Equations (7), (9), and (11) for the slip spring simulations. The imposition of a maximum force allows the slip springs to mostly avoid trapping, and hence the chain can slide through the slip link as it would do if the chain were more highly resolved. However, at the highest Weissenberg numbers, there are a few chains (~1–5) in an ensemble of 100 chains that show trapping even with the maximum entanglement force imposed. The imposition of the maximum slip-spring tension and non-affine motion of the slip-spring is therefore an approximate method of preventing artificial trapping of the slip-link at the kink. We also examined the application of the lower maximum force $F_{\mathrm{ss}}^{\max}=\zeta^{\prime }\dot{\epsilon }L^{2}/32$ on the chain conformation and same results were obtained which are not reported here.

Since the imposition of a maximum entanglement tension in slip springs is ad hoc, we also implement a more physical means of limiting the force on the slip springs, namely performing simulations with a pair of mutually coupled chains. In this method, we generate a pair of chains and equilibrate their conformation independently. After equilibration of the two chains and the application of extensional flow, the motion of a slip-spring anchoring point on one of the chains is coordinated with the motion of the anchor point on another chain, so that the pair moves on average affinely. To reduce the maximum tension on a slip spring, at each time-step, the anchoring point of the less stretched slip-spring (with a lower tension) located on one chain of the pair, is allowed to move farther than the anchoring point of the slip-spring on the other chain of the pair. For example, consider a pair of chains with $N_{s}=32$ where each chain has 8 slip-springs randomly positioned along their contours. We randomly partner each slip-spring of the first chain with a slip link of the second chain. E.g., let us say we partner slip-spring 3 of chain 1 with slip-spring 7 of chain 2. Now, at each time-step, the force in each slip-spring is calculated from the Cohen–Pade equation [18]. Then, the motion of the anchoring points of slip-springs 3 of chain 1 and 7 of chain 2 follows

(12) $$\left\{ \begin{array}{l} If\;F_{3,\;\;SS}^{1,\;t}>F_{7,\;\;SS}^{2,\;t}\to \{\begin{array}{c} {\vec{a}}_{3}^{1,t+\Delta t}={\vec{a}}_{3}^{1,t}+\alpha \Delta t\kappa .{\vec{a}}_{3}^{1,t}\\ {\vec{a}}_{7}^{2,t+\Delta t}={\vec{a}}_{7}^{2,t}+\left(2-\alpha \right)\Delta t\kappa .{\vec{a}}_{7}^{2,t} \end{array}\\ If\;F_{3,\;\;SS}^{1,\;t}<F_{7,\;\;SS}^{2,\;t}\to \{\begin{array}{c} {\vec{a}}_{3}^{1,t+\Delta t}={\vec{a}}_{3}^{1,t}+\left(2-\alpha \right)\Delta t\kappa .{\vec{a}}_{3}^{1,t}\\ {\vec{a}}_{7}^{2,t+\Delta t}={\vec{a}}_{7}^{2,t}+\alpha \Delta t\kappa .{\vec{a}}_{7}^{2,t} \end{array} \end{array}\right.$$

where $F_{3,\;SS}^{1,\;t}$ and ${\vec{a}}_{3}^{1,t}$ are the tension and position vector of the anchor point of slip-spring 3 of chain 1 at time t. The value of the constant α determines the strength of the coupling between entangled kinks, with α=1 giving ordinary affine motion of each anchor point. This algorithm, with α<1 lessens the build-up of tension in the highly stretched slip-spring, allowing the chain to slide more easily through the corresponding slip link, while keeping the average motion of the two coupled slip-springs affine. This method also allows us to implement constraint release in a self-consistent manner, where if a slip-spring on one chain is lost, the coupled slip-spring on the other chain is also removed. No-regeneration of slip links is implemented, consistent with our EKD method, where there exists no entanglement renewal. We repeat the two chains simulations for at least 100 different pairs and average the results.

Thus, we have three alternatives for managing the motion of the slip springs: 1) maximum entanglement force (MEF); 2) two-chain (TC) simulations for four different values of α=0, 0.25, 0.5, 0.75; and 3) affine motion (AM), which corresponds to method 2 above, with α=1 but without the inherent CR of method 2. Our EKD simulations suggest that the tension in slip-springs along the chain should be roughly uniform at the transition strain ($\epsilon =\epsilon_{T}$ or $\tilde{\epsilon }=0$), as shown by the red dashed line in Figure 5. Note that, as explained earlier, the imposition of affine motion on the anchoring points of trapped slip-springs at the kinks results in huge tensions and eventual breakage of slip-springs, even with very small time-steps. Thus, to keep the slip-spring forces bounded for all three methods, whenever the length of a trapped slip-spring becomes greater than 0.99 of its dimensionless maximum length ($N_{k,ss}$), we calculate the tension of that slip-spring for a fixed value $L_{ss}=0.99N_{k,ss}$ and exert this force from the slip-springs on the main chain, independent of the length of the overextended slip spring which might take arbitrarily higher values than its maximum extension. Note that this is only required for alternatives 2 and 3 above where the maximum entanglement force, and relocation of slip-spring’s anchoring point are not applied. In comparison to MEF, where the maximum entanglement force is a function of strain rate, Equation (7), the slip-spring force calculated by considering $L_{ss}/N_{k,s}=\bar{L_{ss}}\;=0.99$ is a constant $F_{ss}^{\bar{L_{ss}}=0.99}=\;111.6$ (in dimensionless units) using the Cohen–Pade force equation in one-dimension [18]. At our high Weissenberg numbers, $Wi_{R}=16,\;32$, the MEF force for long chains will become high, O(100), but as we will discuss in the next section, the modification of the anchoring point’s position in the MEF method at each time step will give the chain time to move towards the slip-spring and relieve most of the generated stress in the slip-spring. The results of slip-spring simulations with these three methods of managing slip-spring motion and tensions are presented in the next section.

## 3. Simulation Results and Discussions

### 3.1. Comparison of Maximum Entanglement Force (MEF), Two-Chain (TC), Affine Motion (AM) Methods

**Figure 9:**
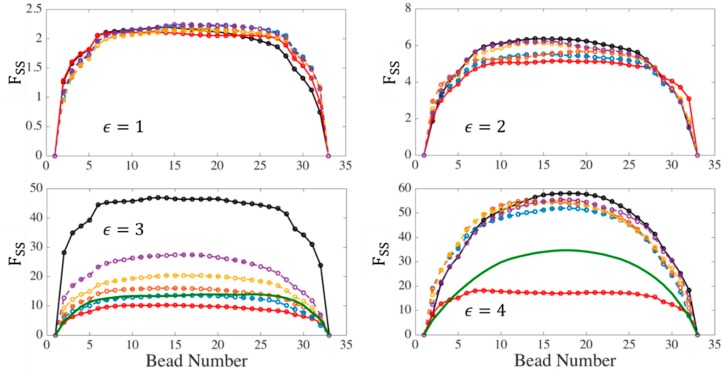
Distribution of tension in slip-springs on a chain with $N_{s}\;=32$ at $Wi_{R}\;=\;32$ at different Hencky strains. Affine motion (AM) results are shown with black lines and maximum entanglement force (MEF) results with red lines. Results for the two-chain (TC) technique are shown with blue, orange, yellow, and purple lines for the strengths of entangled kink coupling given by α=0, 0.25, 0.5, and 0.75 respectively. The solid green line without symbols shows the result of EKD simulations at Hencky strains of 3 and 4. All tensions are averages over an ensemble of 200 chains.

Figure 9 shows the evolution of slip-spring tension along a chain with $N_{s}=32$ and $Z_{0}=8$ at $Wi_{R}=32$ where the effect of trapping becomes important, for all three simulation methods.

At small strains (ϵ=0.01 and 1) the tension distribution is nearly identical in all cases: a nearly uniform tension is found except near the chain ends. However, as the strain increases to around 2–3 Hencky units, the chain starts to align and elongate in the flow direction and the tension under affine motion of slip-spring anchor points diverges from the MEF and TC simulations. At a Hencky strain of ϵ=3, which we will later show is the transition strain at which the kinked state emerges, the tension under affine motion is almost 5 times higher than for the other cases. Up to ϵ=3, both TC and MEF show nearly uniform, moderate, tension; however, as the elongation continues, the tension predicted by the two-chain (TC) method starts to rise faster and approach that for affine motion. We find that at high strain, the coupling technique does not prevent trapping, and the same behavior is obtained as in affine deformation. On the other hand, the tensions obtained for the maximum entanglement force technique remain at much lower values, even for ϵ=4. To compare the tension in slip-spring simulations to that of EKD simulation for the chains used in Figure 9, we perform EKD simulations by exporting the conformation of chains at ϵ=3 from slip-spring simulations and entangling 80% of the kinks (a percentage used in our previous publication for a highly entangled polymer). The results are shown by solid green lines in Figure 9. Note that we are not allowed to use kink dynamics for Hencky strains less than 3, below which the chain has not yet formed the folded state yet. This explains the absence of the green line at ϵ=1 and 2. Comparing EKD with MEF results, it can be seen that although the MEF method keeps the slip-spring tension at moderate values, it predicts lower tension in the chain center than does the EKD simulations of the same chain. However, as noted, both MEF and TC give similar tension distribution at the transition strain ($\epsilon_{T}\sim 3$) which also resembles that obtained from EKD simulations. Thus, the single-chain maximum entanglement force (MEF) method matches that of the TC method up to the transitional strain, where both methods approach that of the EKD algorithm. For higher strains ($\epsilon >\epsilon_{T}$), the dynamics can be tracked by kink dynamics rather than coarse-grained slip-spring simulations. We shall show below, however, the MEF method within the slip spring simulations gives extensional viscosities similar to those of the EKD method from the transitional strain all the way to complete chain extension.

**Figure 10:**
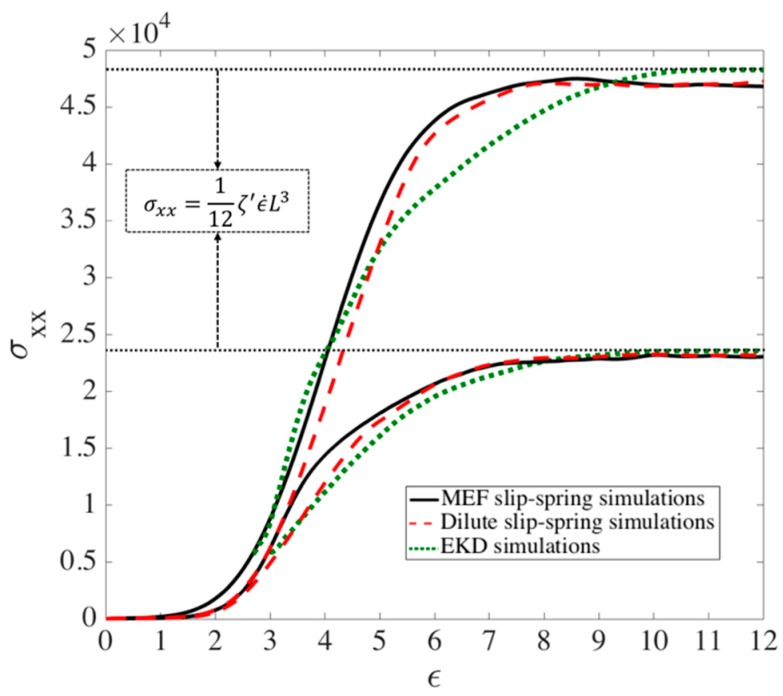
Extensional stress in x-direction ($\sigma_{xx}$) for chains of length $N_{s}=32$ (lower set of curves) and $N_{s}=64$ at $Wi_{R}=32$ (upper set of curves). Black lines are obtained under the MEF assumption while the dashed red lines are for chains without slip-springs; i.e., dilute chains. EKD results, plotted starting from the transition strain ϵ=3, are shown with green dotted lines. The two horizontal dotted lines show the theoretical final stress value that an entangled or unentangled chain will achieve when fully unraveled. The results for both slip link methods and EKD are averaged over an ensemble of 150 chains.

To check the performance of the MEF method beyond the transition strain and during the unraveling regime ($\epsilon >\epsilon_{T}$), a bead-spring chain in a kinked configuration with one kink is generated and a slip-spring with $F_{ss}^{max}=\zeta^{\prime }\dot{\epsilon }L^{2}/8$ is located in the kinked region. A picture of the same chain in the kink dynamics approach is also generated and affine motion is applied to the kink. We study the strain at which the chain achieves full unraveling with both MEF and affine kink dynamics approaches and observe in Appendix B that it takes almost twice the time for the slip-spring simulations with MEF assumption to unravel the chain than it does for the affine motion kink dynamics. It has been previously shown that the chains under EKD also takes longer to unravel, almost twice the strain, compared to affine motion. We also compare in Figure 10 the extensional stress predicted by the EKD model compared to that for the corresponding slip-spring model out to complete extension of polymer. The stress in the slip-spring simulations is calculated using the Kramers–Kirkwood equation for a Rouse chain [60]. The stresses using the EKD simulations are plotted from the transition strains of each chain length, shown by green circles in Figure 10. In the next section, we explain how these transition strains are found. Note that no vertical fitting of EKD results to the slip-spring results is done and the match between EKD and slip-spring stress at the transitional strain is remarkable. The results for the two models are in good agreement except at strains beyond the transitional value. Since very different methods are used in the two models, one involving paired ideally one dimensional chains with no Brownian force (the EKD method) and the other involving slip links with non-affine motion of entanglement points imposed by a maximum force criterion, the agreement of the two methods suggests the robustness of the results. The discrepancy between slip spring simulations and EKD at high strains, ϵ=6 to 8 can be attributed to the discreteness of chains in our bead spring model compared to the resolved kink dynamics approach. We will later show that the stress predicted by the EKD method also gives good agreement with experimental data.

### 3.2. MEF Results for Chain Conformation

Single-chain slip-spring simulations are performed for chains with $N_{s}=16,\;32,\;64,\;128$ springs which corresponds to $N_{k}=N_{s}N_{k,s}\;=80,\;160,\;320,\;640$ Kuhn steps in the chain ($N_{k,s}=5$), respectively. The maximum entanglement force (MEF) method with the value of $F_{\mathrm{ss}}^{\max}=\zeta^{\prime }\dot{\epsilon }L^{2}/8$ is used in all simulations. We seek to determine: (1) the transition strain to the kinked state ($\epsilon_{T}$) as a function of chain length and Weissenberg number; (2) the number of kinks ($N_{\mathrm{Kinks}}$); (3) the ratio of entangled to unentangled kinks ($\rho_{\mathrm{EK}}^{0}$); and (4) the strand length distribution between fold points, with the last three of these determined at the strain at $\epsilon_{T}$. To be consistent with our kink dynamics simulations, no regeneration of slip-springs is considered; therefore, when a slip-spring passes the chain end, it is lost, and no slip springs are re-introduced. As a consequence, the number of slip links, or entanglements, decreases from its initial value, $Z_{0}$, to 0, as the chain becomes completely unraveled. In Section 3.3 we will study the effect of entanglement renewal and constraint release on chain statistics at the kinked state.

**Figure 11:**
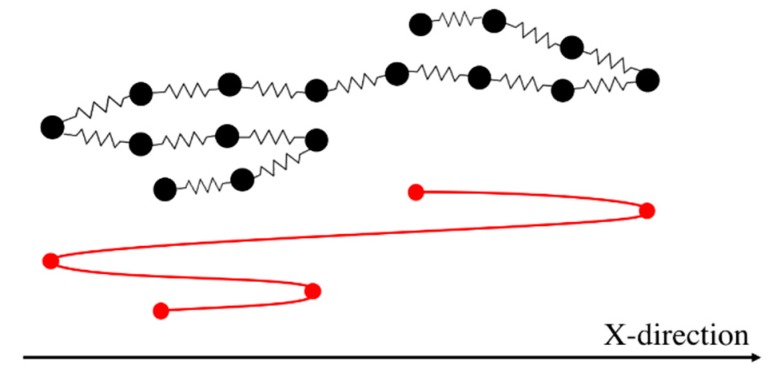
Schematic of mapping of a bead-spring chain into a kinked conformation. The chain has five kinks, including the first and the last beads.

To find the number of kinks, we map the chain along the extension direction (x-axis in our simulations) and find the beads at which the chain folds back on itself, as depicted in Figure 11.

As discussed in our earlier work, to define the kinked state in the slip-spring simulations, the chain should meet two conditions. First, the chain should take on an ‘almost’ one-dimensional conformation, and secondly, the springs on the main chain should be ‘highly stretched’. The first criterion, one dimensionality, is quantified by measuring the ratio of radius of gyration in x-direction to those of the y- and z-directions

(13) $$R_{g_{x/y}}=\frac{R_{g_{x}}}{R_{g_{y}}}=\sqrt{\frac{\langle \sum_{i}{\left(x_{i}-x_{c.m.}\right)}^{2}\rangle }{\langle \sum_{i}{\left(y_{i}-y_{c.m.}\right)}^{2}\rangle }}\;\;\;\;\;,\;\;\;\;\;R_{g_{x/z}}=\frac{R_{g_{x}}}{R_{g_{z}}}=\sqrt{\frac{\langle \sum_{i}{\left(x_{i}-x_{c.m.}\right)}^{2}\rangle }{\langle \sum_{i}{\left(z_{i}-z_{c.m.}\right)}^{2}\rangle }}$$

**Figure 12:**
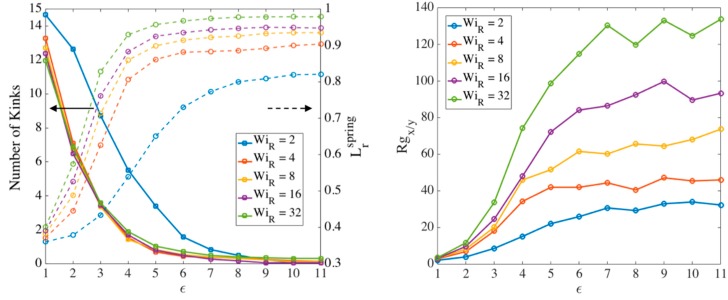
Evolution of (left) number of kinks (left axis) and averaged extension of springs (right axis) and (right) relative radius of gyration ($Rg_{x/y}$) for a chain with $N_{s}=32$ at different Weissenberg from slip-spring simulations with maximum entanglement force (MEF) condition. The averaged extension of springs is calculated using Equation (14).

Here $x_{c.m.}$, $y_{c.m.}$, and $z_{c.m.}$ are x, y, and z components of center of mass of the chain at each time step, respectively. Satisfaction of the second criterion is determined by finding the average relative extension of springs ($L_{r}^{\mathrm{spring}}$) on the main chain. To do so, we calculate the average length of all springs at each time step and then divide that by the maximum extensibility of a spring, which is $N_{k,s}$ in our dimensionless length units.

(14) $$L_{r}^{\mathrm{spring}}=\frac{\frac{1}{N_{s}}\langle \sum_{i=1}^{N_{s}}\left|Q_{i}\right|\rangle }{N_{k,s}}$$

where 〈 .〉 denotes the ensemble average. We choose $L_{r}^{\mathrm{spring}}>0.7$ to be our criterion for ‘nearly fully stretched’ of strands between folds. The rationale behind the choice of 0.7  is given in our previous publication [18]. We plot in Figure 12 the number of kinks and relative radius of gyration $R_{g_{x/y}}$ for a sample chain with $N_{s}=32$ at different Weissenberg numbers. We also plot the average relative extension of springs ($L_{r}^{\mathrm{spring}}$) as the chain is elongated. All the results are ensemble-averaged over 100 chains. These results extend those of our previous work to lower Weissenberg numbers.

**Figure 13:**
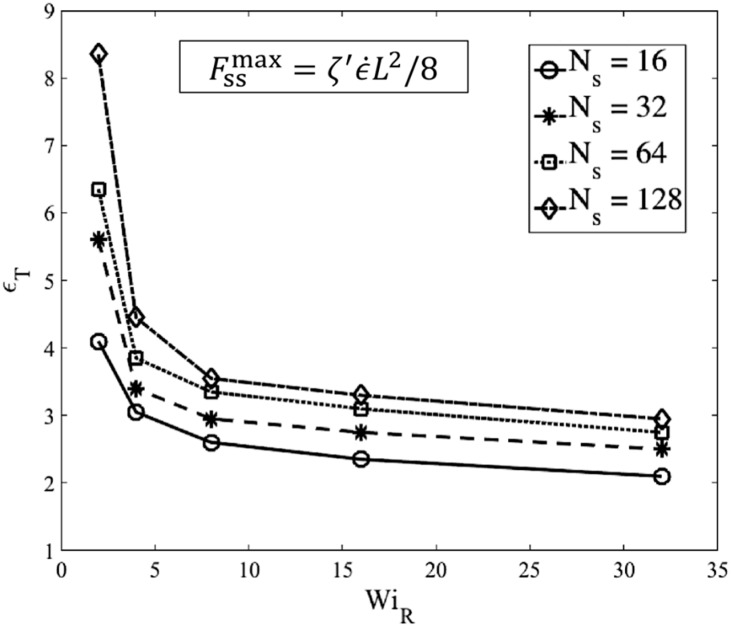
Transition strain for different chain lengths as a function of Rouse Weissenberg number for slip-spring simulations under MEF assumption.

Based on Figure 12, by a Hencky strain of around 3 the chain is almost a one-dimensional object with $Rg_{x}>20Rg_{y}$ for high Weissenberg numbers. At around the same strain, the average extension of springs also reaches the 70% criterion chosen earlier. Based on our observation of chain’s conformation evolution—e.g., Figure 12—we find the strain ($\epsilon_{T}$) at which the two criteria (nearly 1D configuration and average spring stretch of 70%) are met and plot the results for different chain lengths in Figure 13.

**Figure 14:**
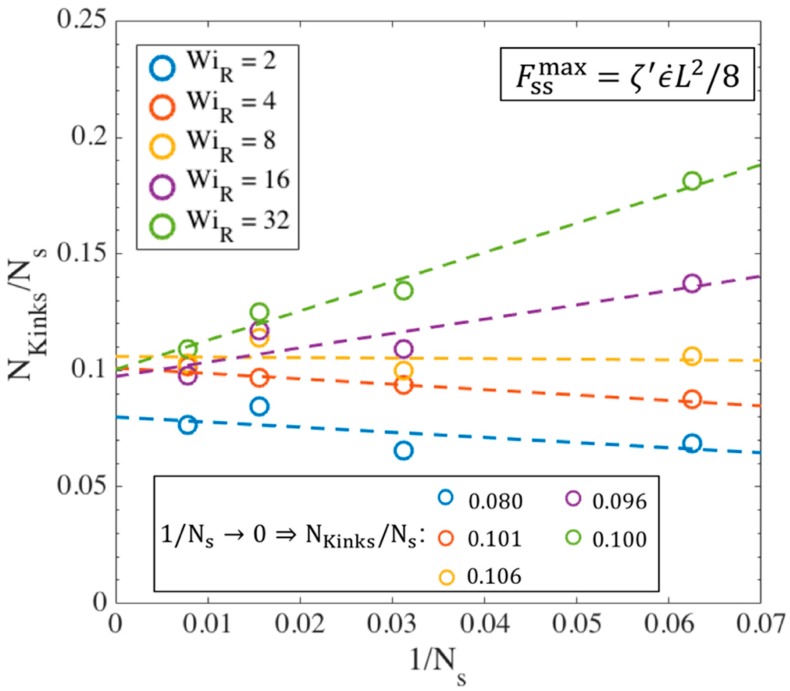
Number of kinks divided by the number of springs $N_{s}$ plotted against $1/N_{s}$ for different values of $Wi_{R}$. The intercept with the y-axis is the asymptotic value of the ratio $N_{Kinks}/N_{s}$ given in the legend for each $Wi_{R}$.

Figure 13 shows that the transition strain ($\epsilon_{T}$) for different chain lengths approach a value of around 3 at high Weissenberg numbers. This analysis confirms and strengthens our previous estimate of a transition strain at around $\epsilon_{T}\sim 3$ [18]. Figure 14 shows the number of kinks on the chain at the transition strains found in Figure 13.

A linear correlation $N_{\mathrm{Kinks}}=wN_{s}$, is observed between the number of kinks and number of springs, with the ratio w converging to 0.1 at high values of $Wi_{R}$. This is close to the value obtained in our previous publication where we counted the number of kinks only at $\epsilon_{T}=3$ for all cases, while here we count the number of kinks at the strains corresponding to the onset of the kinked state at each Weissenberg number for each chain length. As explained before, $N_{s}$ and $N_{k,s}$ can be related to the molecular weight of a specific polymer based on its characteristic ratio $C_{\infty }$ [61].

We also determine the ratio of entangled to unentangled kinks at the transition to the kinked state. We consider an ‘entangled kink’ to be one for which there is a slip-link in a ‘folded region’, which consists of the two springs on either side of a bead located at a ‘fold point’, which is a bead for which the two neighboring beads are both either at small or larger values of x, the stretch direction. If multiple slip-links are within the same kinked region, the kink only counts as a single ‘entangled kink’. We then compute the ratio of entangled to total kinks as

(15) $$\rho_{EK}^{0}=\frac{N_{\mathrm{Kinks}}^{\mathrm{Entangled}}}{N_{\mathrm{Kinks}}^{\mathrm{Total}}}$$

**Figure 15:**
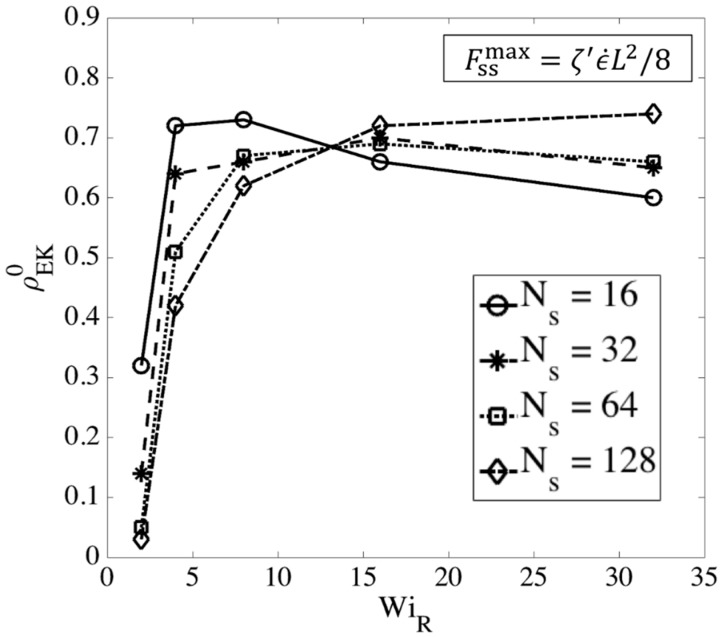
Fraction of entangled to total kinks ($\rho_{EK}^{0}$) for different chain lengths as a function of Weissenberg number at the transition to the kinked state.

The superscript (0) is used because the entangled kink density is calculated at the transition strain determined from the slip-spring simulations; this value, given by Equation (15), is used as the initial entanglement fraction of kinks in the kink dynamics (EKD) simulations which start at $\tilde{\epsilon }=0$. Figure 15 shows the ratio of the numbers of entangled to total kinks for different chain lengths obtained from slip-spring simulations, with no slip-spring regeneration or constraint release.

Figure 15 shows that at high Weissenberg numbers, for all chain lengths, the fraction of entangled kinks converges to the range [0.6−0.7]. We had the value $\rho_{EK}^{0}=0.8$ in kink dynamics simulations for highly entangled polystyrene samples in our previous publication [18]. Based on the results in Figure 15, however, we here choose $\rho_{EK}^{0}=0.65$ as a more appropriate value for EKD simulations. We note that this value is appropriate for entangled polymer solutions of volume fraction 0.2, but may well differ for other volume fractions. Different number of Kuhn steps between slip-links would be needed to explore further the dependencies of $\rho_{EK}^{0}$ on polymer concentration.

**Figure 16:**
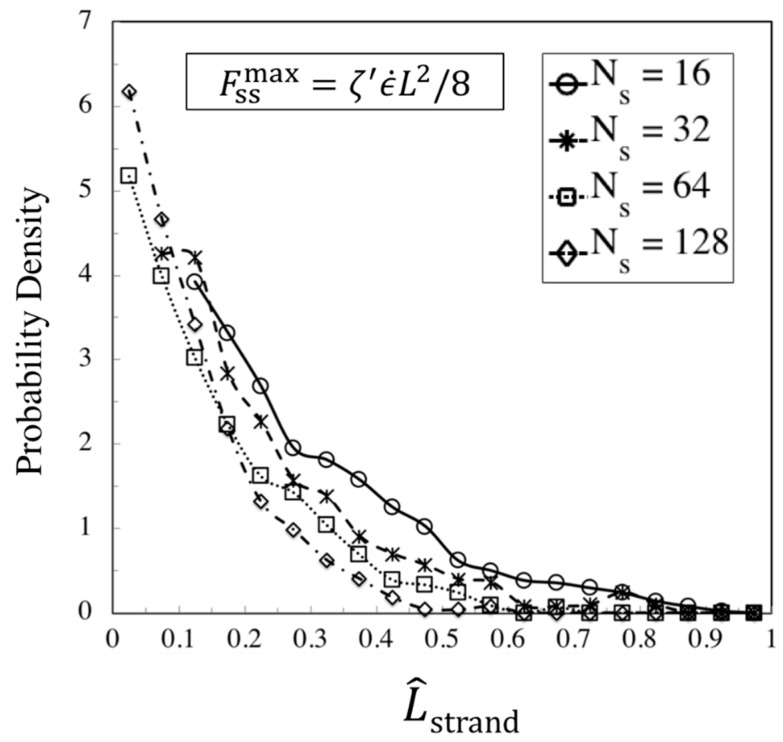
Strand length probability density for different chain lengths at $Wi_{R}=32$. Strand lengths are obtained at the transition strain corresponding to formation of the kinked state of each chain at $Wi_{R}=32$.

Finally, the strand length distribution at the transition to the kinked state is found by measuring the lengths of strands between fold points (beads located at the kinks). Strand lengths normalized by the contour length of the chains are plotted in Figure 16 for $Wi_{R}=32$, which is our strongest flow. The distribution function is approximately exponential. Results for $Wi_{R}=16\;$and 8 were similar, except that there are significant deviations from an exponential form. Using the MATLAB toolbox, we fit to an exponential density function for the two longest chains ($N_{s}=64$ and 128), and find

(16) $$f\left({\hat{L}}_{\mathrm{strand}}\right)=\left(1/\mu \right)\exp\left(-{\hat{L}}_{\mathrm{strand}}/\mu \right)\;,\;\mu =0.193$$

We summarize the above results with the following formulas for the statistics of a chain with arbitrary length (or molecular weight) at its kinked state produced at asymptotically high strain rates:

1. $\epsilon_{T}≅3$
2. $N_{\mathrm{Kinks}}≅0.02N_{k}\;\left(e.g.,\;\mathrm{for}\;\mathrm{polystyrene}\;N_{k}=M_{w}/742\right)$ [18]
3. Fraction of entangled kinks: $\rho_{EK}^{0}≅65\%$
4. Strand length probability distribution: $f\left({\hat{L}}_{\mathrm{strand}}\right)=\frac{1}{\mu }\exp\left(-\frac{{\hat{L}}_{\mathrm{strand}}}{\mu }\right)\;,\;\mu =0.193$

### 3.3. Addition of Slip-Link Regeneration and Constraint Release to Slip-Link Model

Finally, we analyze the effect of slip-spring regeneration and constraint release (CR). Previously, Likhtman [41] and Del Biondo et al. [43] implemented constraint release by partnering slip-links between different chains within an ensemble of chains, in simulations of linear viscoelasticity and simple shear, respectively. In their method, when a slip-link passes off the end of its chain, it is destroyed, and, simultaneously, the partner slip link on the partner chain is destroyed. Then two new slip links are generated, one at the end of a randomly chosen chain and the other one on a random position along the contour length of another chain. This results in a constant number of slip-springs at all times, even during a deformation. In his single-chain simulations, Uneyama [29] defined destruction and creation probabilities following Glauber-type dynamics [62] and showed a reduction in the number of slip-links (Z) with time after start-up of shear flow [33]. A similar reduction under shear flow was also reported by Kushwala and Shaqfeh [63] and by Schieber and coworkers [45]. Here, we implement a simple single-chain technique to incorporate the addition/removal of slip-springs without needing an ensemble of chains or a partnering of slip-links on different chains. Although our method of regeneration and CR is not as detailed as in previous studies, the method targets some limiting cases. Four different conditions are considered:

1. Regeneration off—CR off: when a slip-link passes through its chain end, it is destroyed, and no further action is taken for other slip-links on the chain. This results in a continual reduction in the number of slip-links until all of them are gone. This condition has been used to obtain the results in Section 3.2.
2. Regeneration off—CR on: when a slip-link passes through its chain end, it is destroyed. Simultaneously, another slip-link on the same chain is randomly chosen and removed to represent constraint release produced by other chains, which are not simulated directly. Since there is no regeneration, slip-springs disappear faster than in Condition 1 above.
3. Regeneration on—CR off: If a slip-link passes by the chain end, it is removed and instantaneously recreated at a random location on the chain. Thus, the total number of entanglements stays constant under this condition.
4. Regeneration on—CR on: If the chain end passes through a slip-link, the slip-link is destroyed and recreated at a random position on the same chain. Simultaneously, another randomly chosen slip-link is removed and recreated at a random position along the chain. Therefore, the number of slip-links stays constant.

**Figure 17:**
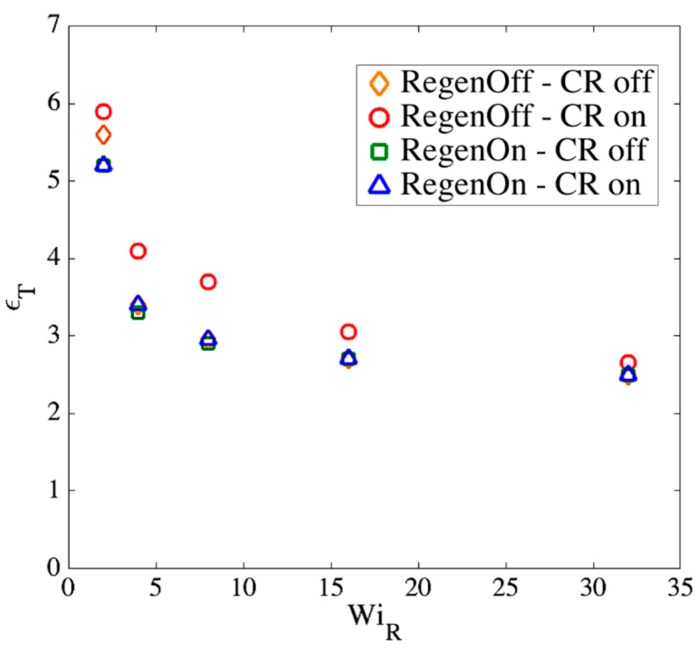
Transition strain ($\epsilon_{T}$) as a function of Rouse Weissenberg number ($Wi_{R}$) for a chain with $N_{s}\;=32$ springs under different renewal/CR algorithms. A constant value of $F_{ss}^{max}$ is used for all four conditions.

**Figure 18:**
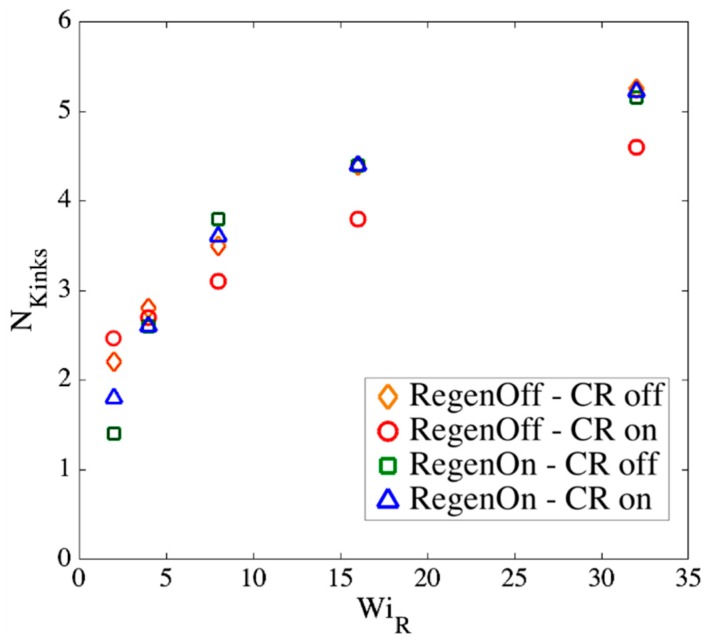
The same as Figure 15, except for the number of kinks ($N_{Kinks}$).

**Figure 19:**
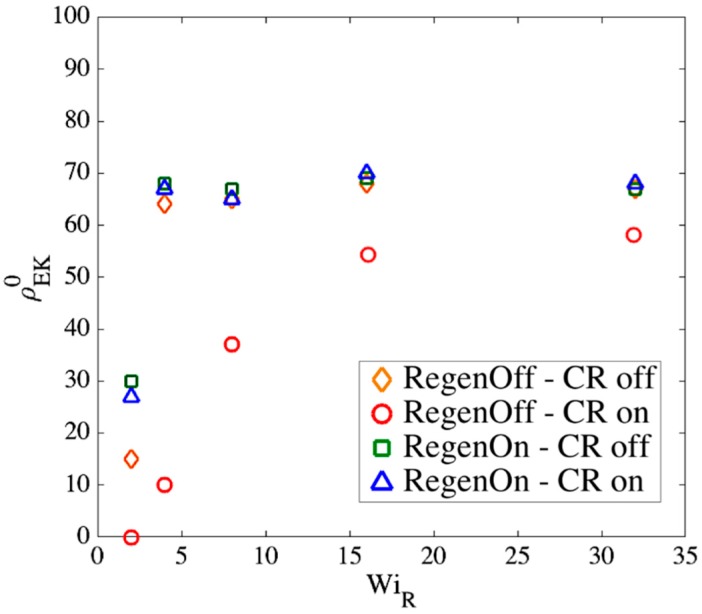
The same as Figure 15, except for the entanglement density ($\rho_{EK}^{0}$).

Note that the actual constraint release dynamics should lie somewhere between these extreme conditions; however, we now show that our results are almost independent of these assumptions at high $Wi_{R}$. Figure 17, Figure 18 and Figure 19 show the effect of our regeneration/CR algorithms on the transition strain, the number of kinks, and the entanglement density for a chain with $N_{s}=32$ ($Z_{0}=8$), respectively. Similar responses are obtained for other chain lengths, which we do not show here. The maximum entanglement force of $F_{ss}^{max}=f_{CHC}=\zeta^{\prime }\dot{\epsilon }L^{2}/8$ is used for all cases.

According to Figure 17, Figure 18 and Figure 19, at high Rouse Weissenberg numbers, the responses become nearly identical for the different cases, showing near independence of the results from the constraint release or regeneration processes at high $Wi_{R}$. Figure 17 shows that at lower $Wi_{R}$, there are differences; for example, with ‘Regeneration on’ (i.e., a constant number of slip-springs at all strains), the kinked state is attained faster, but results converge as $Wi_{R}\;$increases. The same convergence at high $Wi_{R}$ is obtained for the number of kinks, Figure 18, except for ‘Regeneration off—CR on’ where the number of kinks is, perhaps not surprisingly, somewhat less than for the other three conditions. The entanglement density at the onset of the kinked state also shows the same convergence at high $Wi_{R}$; see Figure 19. Interestingly, $\rho_{EK}^{0}$ under ‘Regeneration off—CR on’ is close to that obtained under the other conditions at high Weissenberg numbers; one might expect that with the rate of loss of slip springs doubled due to CR, and no slip springs regenerated, the density of entangled slip springs would be much reduced relative to the other cases. The behavior observed is explained by considering that first of all, at high $Wi_{R}$, there are fewer kinks under ‘Regeneration off—CR on’ as shown in Figure 18, and since $\rho_{EK}^{0}$ is the fraction of kinks that have a slip- link, the reduction in kinks tends to offset the decrease in number of slip links. Also for this case, the fewer slip-springs that do remain tend to accumulate at the kinks, thus keeping the percentage of kinks with a slip link relatively high despite the fewer slip springs present. Thus, the entanglement percentage is close to that of the other algorithms. At high $Wi_{R}$, since the chain quickly aligns and stretches in the flow direction, by the time the initial slip-springs start to leave the chain by passing off the chain ends, the kinked state has been achieved, and renewal of slip-springs does not change the conformation. The newly added slip-links, under conditions 3 and 4 above, will be quickly convected to chain ends or existing folded regions. As mentioned before, the accumulation of newly generated entanglements (i.e., slip springs) at already entangled kinks does not change the entanglement percentage. Although the correct renewal algorithm lies between our arbitrary assumptions for CR and Regeneration of slip-springs, we see that at high $Wi_{R}$, the results under all renewal assumptions converge to the same values. Note that this independency of the results on the process of convective constraint release (CCR) is only valid for strong extensional flows where the chain conformation quickly falls into the folded region. The same is not true for shear flows, for example, where the regeneration of slip-springs, or entanglements in general, affect chain conformation and tumbling becomes important.

In the next section, we use the $\epsilon_{T}$, $N_{Kinks}$, $\rho_{EK}^{0}$ and ${\hat{L}}_{\mathrm{strand}}$ values obtained by the slip-spring simulations as initial conditions in our entangled kink dynamics algorithm (EKD) and compare the predictions of the kink dynamics model to experimental data.

### 3.4. Comparison of Kink Dynamics Results with Experimental Data

Having used slip-spring simulations to obtain the details describing the conformational and entanglement state of polymer chains as they first enter the kinked state, we now perform EKD simulations using these realistic starting conditions. First, we determine the number of kinks for a specific molecular weight based on the linear correlation we found in Section 3.2. Then, we use the exponential distribution to specify the strand lengths between the kinks. After generating the kinked conformation based on the average strand length and the number of kinks, we put the chains into a simulation box and mutually entangle 65% of the kinks. Previously, we used an 80% entanglement density to represent a highly entangled polymer. All results obtained with the EKD simulations are shifted to the Hencky strain of 3 which we showed is approximately the onset of the kinked state.

**Figure 20:**
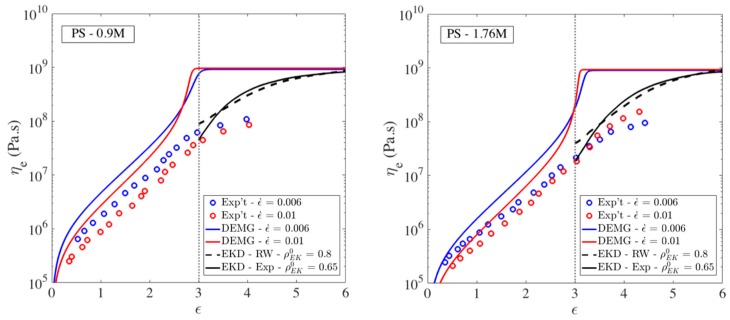
Comparison of uniaxial extensional experimental data [22,49] (o) for 33 wt % 0.9M polystyrene (PS) (left) and 18 wt % 1.76M PS with predictions of the DEMG model and the entangled kink dynamics (EKD) model using the random walk (RW) model for the initial distribution of strand lengths and $\rho_{EK}^{0}=0.8\%$ (dashed line) and using the exponential strand length distribution (Exp) with $\;\rho_{EK}^{0}=0.65$ (solid line). The EKD predictions are in both cases plotted starting from the transition strain of $\epsilon_{T}=3$.

The results are shown in Figure 20. In addition to experimental data shown with symbols, we have added the predictions of Doi-Edwards-Marrucci-Grizzuti (DEMG) model which has the segmental stretch added to the basic Doi-Edwards theory [61]. We have shown in previous work that including CCR in the tube model does not improve the predictions of the tube model for these samples [18]. Figure 20 also shows that the evolution of extensional viscosity predicted here using the exponential distribution of strand lengths and $\rho_{EK}^{0}=0.65$ is not much different from that obtained in our previous work, which used a random walk (RW) generator to obtain the initial strand length distribution with $\rho_{EK}^{0}=0.8$ of the kinks initially entangled. Since the transition strain ($\epsilon_{T}$) and linear correlation between chain length and number of kinks is the same as our previous publication, the main differences in our new data is in the entanglement density and strand length distribution. A random walk chain conformation generator results in a similar, nearly exponential strand length as our exponential distribution derived from the slip-spring simulations, which explains the similar trend of data (solid and dashed black lines), as shown in Figure 20. Note that unlike the tube theory prediction with an inflection point at Hencky strain of ϵ~3, where the solid line changes from a concave-up to convex-up curvature, both the experimental data and EKD predictions exhibit a convex-up curvature over the entire range of high strains.

In Figure 20, there are small differences in the initial viscosity and in the rate of rise of the viscosity at strains above $\epsilon_{T}=3$, between results from our new simulations (solid lines) and our previous EKD simulations (dashed lines), which can be attributed, respectively, to the lower value of initial percentage of entangled kinks and to the exponential distribution of strands, in our new results. Notice that we used identical transitions strain ($\epsilon_{T}=3$) and the same linear relationship between the number of kinks and the number of Kuhn steps in the chain, as used before [18].

## 4. Conclusions and Future Directions

We used both modified single-chain slip-spring simulations and multi-chain entangled kink dynamics techniques to study the extensional rheometry of long linear chains at high strains and high Rouse Weissenberg numbers $Wi_{R}$. The slip-spring simulations gave the configurations and entanglement density of a linear polymer chain at the transition to the kinked state, before unraveling starts to dominate the behavior. To perform these slip-spring simulations under fast extensional flows, we imposed a maximum slip-spring force to prevent permanent trapping of slip links at nascent fold points along the chain, with force set by a simple analysis of the force at an entangled kink between two symmetrically hooked chains. We also carried out slip-spring simulations with the slip springs on one chain paired randomly with those on a second chain and allowed less-than-affine motion of the slip spring with high tension to be offset by super-affine motion of the partner slip spring so as to create affine motion on average. We found that this method of limiting the tension of the slip springs produced similar slip-spring tension distributions as the maximum force method, up to the formation of the kinked state. The results show that multiple methods of preventing overly high tensions on the slip springs produce similar results up to the formation of the kinked state. The effect of slip-spring renewal and constraint release on the number of kinks $N_{\mathrm{kinks}}$, fraction of kinks that are entangled $\rho_{EK}^{0}$, and transition strain to the kinked state $\epsilon_{T}$ were found to converge at high $Wi_{R}$ to similar values regardless of the constraint release and constraint renewal assumptions used in the slip-spring model. Using the number of entangled and unentangled kinks and their spacing along the chain predicted by the slip-spring model at the onset of the kinked state as initial conditions, entangled kink dynamics (EKD) simulations were carried out and the predicted stress growth was found to be similar to that obtained from slip-spring simulations with the maximum slip-spring force, up to full unraveling of the chain. The EKD simulations for long polystyrene chains at high Weissenberg numbers gave stresses that compared favorably with experimental data. Thus, combining slip-spring simulations with entangled kink dynamics simulations provide a complete picture of the dynamics of extension of linear chains from equilibrium to the completely unfolded state. The results also suggest that the single chain slip-spring simulation technique, with a maximum slip-spring force and non-affine motion of anchoring points, can be applied to other nonlinear flows, such as shear flows, at high Weissenberg numbers. Developing an appropriate maximum force criterion for shear and a reliable convective constraint release technique, which in shear flows become very important due to the tumbling phenomena of the chain, is an important future direction.

## Figures and Tables

**Figure B1:**
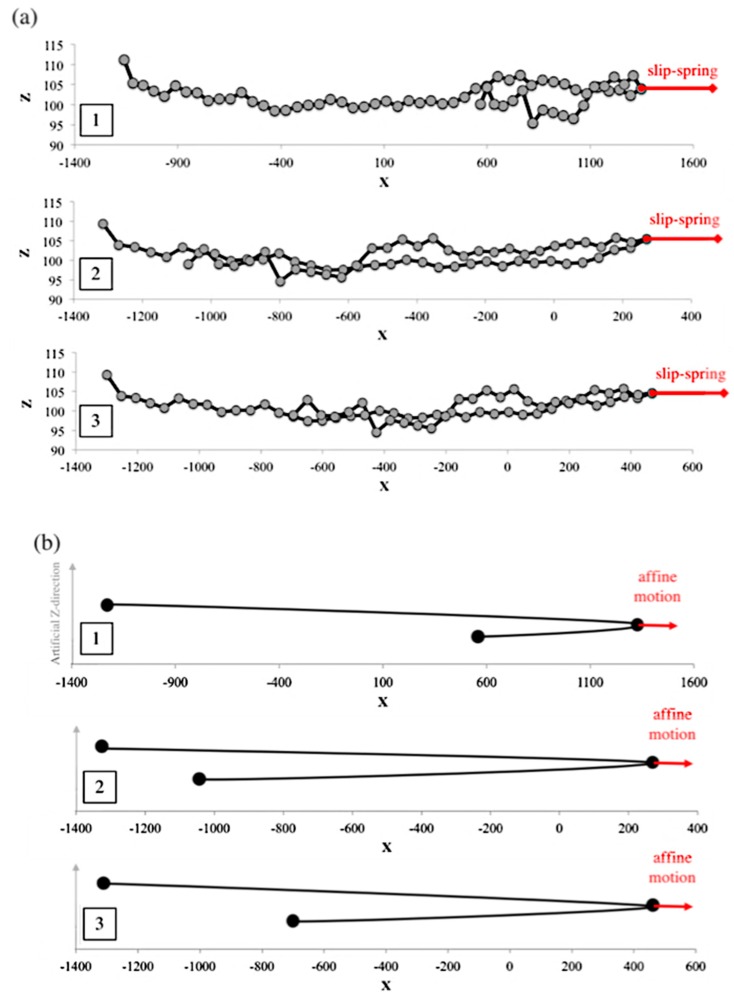
Three conformations used to compare the unraveling behavior of a chain from the kinked to fully unraveled state in (**a**) the slip-spring simulations and (**b**) kink dynamics simulations. In the slip-spring simulations, $N_{s}=64$ and the maximum applied force, $F_{ss}^{max}=\zeta^{\prime }\dot{\epsilon }L^{2}/8$, is applied. In the kink dynamics simulations, affine motion is imposed at the kink.

**Table 1:** Evolution equations for vector **x** of kink positions under dilute and affine assumptions.

	Position Evolution Matrix A : dx/dt=A.x	$\mathrm{Stress}:\;\sigma_{xx}$
Dilute	$\frac{1}{4}\dot{\epsilon }\left[\begin{array}{ccccccccccc} 2 & 2 & 0 & 0 & 0 & . & . & . & . & . & .\\ 1 & 2 & 1 & 0 & 0 & . & . & . & . & . & .\\ 0 & 1 & 2 & 1 & 0 & . & . & . & . & . & .\\ . & . & . & . & . & . & . & . & . & . & .\\ . & . & . & . & . & . & 0 & 1 & 2 & 1 & 0\\ . & . & . & . & . & . & 0 & 0 & 1 & 2 & 1\\ . & . & . & . & . & . & 0 & 0 & 0 & 2 & 2 \end{array}\right]$	$\frac{1}{12}\nu \zeta^{\prime }\dot{\epsilon }\langle \sum_{i=1}^{N_{\mathrm{Kinks}}}{\left|x_{i}-x_{i-1}\right|}^{3}\rangle$
Affine	$\dot{\epsilon }I$	$\nu \langle \sum_{i=1}^{N_{\mathrm{Kinks}}}\left|\begin{array}{c} -\frac{1}{6}\zeta^{\prime }\dot{\epsilon }{\left(x_{i}-x_{i-1}\right)}^{2}\left(x_{i}+2x_{i-1}\right)\\ +\zeta^{\prime }V_{i}\frac{{\left(x_{i}-x_{i-1\;}\right)}^{2}}{2}+f_{i-1}\left(x_{i}-x_{i-1}\right) \end{array}\right|\rangle$

**Table 2:** Input parameters for slip-spring simulations.

Input	$N_{s}$	$N_{k,s}$	$N_{e}$	$N_{k,ss}$	$\zeta_{s}$	$Wi_{R}$
Value	16, 32, 64, 128	5	4	2.5	0.1	2,4,8,16,32

## Appendix A

### Time-Step Size in Slip-Spring Simulations

In our slip-spring simulations using MEF technique, the choice of $\zeta_{s}$, which is the friction coefficient of the slip links along the polymer chain’s contour length, is very important, since it determines the escape time of an slip link from the chain, as mentioned by Likhtman [41]. Here, we develop a technique to determine the ratio of $\zeta_{s}/\Delta t$, where Δt is the size of the time step in our slip-spring simulations. Upon convection of a slip link to a kinked region, which is composed by three beads, Figure 8, the slip link position on the chain fluctuates along the length of the two springs composing the folded region, based on Equation (14). As the length, and consequently the generated force, of a slip-spring in the kinked region increases, the displacement of the slip link position at each time step increases, which may result in displacing the slip link position beyond one spring at a single time step, artificially making an entangled kink unentangled. In the absence of any form of constraint release, the only way that an entangled kink can disappear on a chain is that the chain destroys that kink, either by passing its ends through the kink or by shrinking the length of a nearby strand length. Therefore, a slip-spring at a kink should not be able to escape the kinked region because of input simulation parameters such as $\zeta_{s}$ or size of the time-step, Δt. So, from t to t+Δt, the displacement Δx of the slip-link along the spring should not exceed a certain value (found below) that artificially moves the slip-link out of the kinked region without the chain needing to slide through the slip link. If Δx becomes too large the slip link will move past one than one bead, and the slip-spring gets out while the kink is not unraveled yet. This picture helps us determine the relationship between time-step size, Δt, and friction coefficient of slip-links on the chain, $\zeta_{s}$. Based on the evolution equation for $x_{j}$, Equation (14), the maximum value of Δx during a single time-step Δt for a slip-link is achieved when the slip-spring force ($F_{j}^{SS}$) and neighboring beads’ connecting vector (${\vec{r}}_{\lfloor x_{j}\rfloor +1}-{\vec{r}}_{\left\lfloor x_{j}\right\rfloor }$) are at their maximum values, which we can estimate by a one-dimensional approximation

(A1) $$\left\{ \begin{array}{l} 1-D\;\mathrm{limit}\to {\vec{r}}_{\lfloor x_{j}\rfloor +1}-{\vec{r}}_{\left\lfloor x_{j}\right\rfloor }=X_{\lfloor x_{j}\rfloor +1}-X_{\left\lfloor x_{j}\right\rfloor }\overset{max}{\to }N_{k,s}\\ 1-D\;\mathrm{limit}\to F_{ss}^{max}=\frac{1}{8}\zeta^{\prime }\dot{\epsilon }{\left(N_{s}N_{k,s}\right)}^{2} \end{array}\right.\Rightarrow \Delta x=\left(\frac{\zeta^{\prime }\dot{\epsilon }N_{s}^{2}N_{k,s}^{3}}{8}\right)\frac{\Delta t}{\zeta_{s}}+\sqrt{\frac{2\Delta t}{\zeta_{r}}}n$$

where X is the x-component of the position vector $\vec{r}$ of a bead and (n) is a random number between −1 and 1. Based on the position of the slip-link along a spring on the main chain in the kinked region, the Δx value in a single time-step should be prevented from moving across more than one bead. The criteria are summarized in Table A1.

**Table A1:** Criteria for maximum Δx and corresponding times step Δt for two locations of slip-link in the kinked region.

Condition	Direction of Slip-Spring Jump	Criterion for Δx
i		$\Delta x\leq \lfloor x_{j}\rfloor -x_{j}+2$ $\left(\frac{\zeta^{\prime }\dot{\epsilon }N_{s}^{2}N_{k,s}^{3}}{8}\right)\frac{\Delta t}{\zeta_{s}}+\sqrt{\frac{2\Delta t}{\zeta_{s}}}n\leq \lfloor x_{j}\rfloor -x_{j}+2$
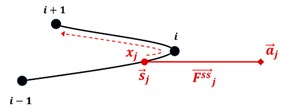
ii		$\Delta x\geq \lfloor x_{j}\rfloor -x_{j}-1$ $\left(\frac{\zeta^{\prime }\dot{\epsilon }N_{s}^{2}N_{k,s}^{3}}{8}\right)\frac{\Delta t}{\zeta_{s}}+\sqrt{\frac{2\Delta t}{\zeta_{s}}}n\geq \lfloor x_{j}\rfloor -x_{j}-1$
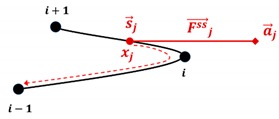

Here we perform an analysis for condition (i) of Table A1, that can be extended to condition (ii) as well. For the first condition, we simplify Equation (A1) by considering $\sqrt{\frac{\Delta t}{\zeta_{s}}}$ as the variable of the equation and replacing the coefficients of Equation (A1) with A, B, C, as

(A2) $$\left\{\begin{array}{l} A=\left(\frac{\zeta^{\prime }\dot{\epsilon }N_{s}^{2}N_{k,s}^{3}}{8}\right)\\ B=n\sqrt{2}\;\;\;\;\;\;\;\;\;\;\;\;\;\;\;\;\\ C=\lfloor x_{i}\rfloor -x_{i}+2 \end{array}\right\}\Rightarrow A{\left(\sqrt{\frac{\Delta t}{\zeta_{s}}}\right)}^{2}+B\left(\sqrt{\frac{\Delta t}{\zeta_{s}}}\right)-C\leq 0$$

By definition, we have following criteria for the parameters in the inequality A2

$$\{\begin{array}{c} A>0\;\\ -\sqrt{2}<B<\sqrt{2}\\ C>0\;\\ \sqrt{\frac{\Delta t}{\zeta_{s}}}>0\; \end{array}$$

To satisfy the inequality A2, two conditions should be met: (1) the discriminate of the equation ($A{\left(\sqrt{\Delta t/\zeta_{s}}\right)}^{2}+B\left(\sqrt{\Delta t/\zeta_{s}}\right)-C=0$) should be positive, $\Delta =B^{2}+4AC>0\;$and (2) the variable $\sqrt{\Delta t/\zeta_{s}}$ should take values within the two roots of the equation ($A{\left(\sqrt{\Delta t/\zeta_{s}}\right)}^{2}+B\left(\sqrt{\Delta t/\zeta_{s}}\right)-C=0$), $P_{1}\leq \sqrt{\Delta t/\zeta_{s}}\leq P_{2}$, where $P_{1}$ and $P_{2}$ are the two roots, given by

$$\{\begin{array}{c} P_{1}=\frac{-B-\sqrt{B^{2}+4AC}}{2A}<0\\ P_{2}=\frac{-B+\sqrt{B^{2}+4AC}}{2A}>0 \end{array}$$

Since 4AC term in above expressions is always positive, for any value of B between $-\sqrt{2}$ and $\sqrt{2}$, numerator of $P_{1}$ will be negative, which results in an always negative $P_{1}$ as the smaller root of the quadratic equation. However, the value of $\sqrt{\Delta t/\zeta_{s}}$ is always non-negative. Thus, the final bounds for $\sqrt{\Delta t/\zeta_{s}}$ to satisfy the inequality A2 are

(A3) $$0\leq \sqrt{\frac{\Delta t}{\zeta_{s}}}\leq \frac{-B+\sqrt{B^{2}+4AC}}{2A}=P_{2}$$

Based on Equation (A3), we have to make sure that our choice of time step size and $\zeta_{s}$ give us a $\sqrt{\Delta t/\zeta_{s}}$ less than $P_{2}$. However, the value $P_{2}$ can change based on B and C which themselves can vary based on the random number generator and location of slip-link, respectively. However, if for the smallest value of $P_{2}$, our choice of $\sqrt{\Delta t/\zeta_{s}}$ still meets A3 criterion, we can make sure that for any generated random number or slip-link location on the chain, A3, and consequently A2 are satisfied. The smallest value of $P_{2}\;$is achieved in the limit of $B=\sqrt{2}$ and C=1. A is calculated from the chain length (i.e., the number of Kuhn lengths per spring, $N_{k,s}$ and number of springs per chain, $N_{s}$), the applied strain rate, $\dot{\epsilon }$, and the friction coefficient per unit length, $\zeta^{\prime }$, where the latter is given by Equation (14). Table A2 shows the values of $P_{2}^{2}$ for the choice of $B=\sqrt{2}$ and C=1, for different chain lengths at difference Weissenberg numbers.

**Table A2:** Values of $P_{2}^{2}$ corresponding to the upper limit of $\Delta t/\zeta_{s}$ in the slip-spring simulations for various chain lengths and Rouse Weissenberg numbers.

	$N_{s}=16$	$N_{s}=32$	$N_{s}=64$	$N_{s}=128$
$Wi_{R}=2$	0.0128	0.012	0.01181	0.01165
$Wi_{R}=4$	0.00674	0.0063	0.0063	0.0061
$Wi_{R}=8$	0.00347	0.0033	0.00326	0.00316
$Wi_{R}=16$	0.00178	0.00173	0.00164	0.00162
$Wi_{R}=32$	0.00091	0.00086	0.00084	0.00081

Based on $P_{2}^{2}$ values in Table A2, the choice of $\Delta t=10^{-4}$ and $\zeta_{s}=0.1$, which corresponds to $\Delta t/\zeta_{s}=10^{-3}$, is justified for all chain lengths up to $Wi_{R}=16$. For $Wi_{R}=32$, we reduce the time step size and choose $\Delta t=10^{-5}$ and $\zeta_{s}=0.1$, corresponding to $\Delta t/\zeta_{s}=10^{-4}$, to make $\Delta t/\zeta_{s}$ less than the values of the last row of Table A2. Since simulations are performed with explicit time-integration, possible spring breakage and slip-spring force in excess of the maximum value are also checked at each time step. Applying the same analysis to condition (ii) results in the same bounding values for Δt. Note that using an implicit technique may remove the need of such an analysis of maximum timestep to avoid the slip link moving past more than one bead, or the spring breaking, or the maximum force being exceeded. However, an implicit method for single-chain slip-spring simulations is not yet available, and having the artificial friction coefficient, $\zeta_{s}$ present in the slip-spring model makes above analysis for timestep size necessary. In deriving Δt, we imposed the maximum slip-spring force $F_{\mathrm{ss}}^{\max}=\zeta^{\prime }\dot{\epsilon }{\left(N_{s}N_{k,s}\right)}^{2}/8$; therefore for any slip-spring force below this, the conditions (i) and (ii) are satisfied if $\Delta t/\zeta_{s}$ is constrained as specified in Table A2.

## Appendix B

### MEF Performance in Unraveling a Kinked Chain

To compare the MEF method with kink dynamics simulations, we generate a bead-spring chain in three different folded states with one slip-spring at the kink, as shown in Figure B1a. The slip-spring force is at its maximum $F_{ss}^{max}=\zeta^{\prime }\dot{\epsilon }L^{2}/8$ at the start of the slip spring simulations and we keep this force at this constant maximum value for the slip-springs in these calculations. We also perform kink dynamics simulations under affine motion for the three conformations, as illustrated Figure B1b, and then compare the Hencky strain at which fully unraveled (FU) chain is achieved in both slip-spring and kink dynamics simulations, which we label $\epsilon_{SS}^{FU}$ and $\epsilon_{Kink}^{FU}$, respectively. Table B1 shows the values of $\epsilon_{SS}^{FU}$, $\epsilon_{Kink}^{FU}$ and their ratios for the three conformations.

**Table B1:** Hencky strains at which the slip-spring and kink conformations of Figure B1 achieve a fully unraveled state.

Conformation	$\epsilon_{SS}^{FU}$	$\epsilon_{Kink}^{FU}$	$\epsilon_{SS}^{FU}/\epsilon_{Kink}^{FU}$
1	1.22	0.61	2.00
2	4.87	2.42	2.01
3	2.99	1.52	1.97

For all three cases, twice as much strain is required to fully unravel the chain from the kinked state in the slip-spring simulations than under the affine motion assumption. The factor of two can be attributed to the temporary trapping of the slip-spring between the two springs that compose the kinked region. Since the slip-spring spends half of its lifetime on the top strand and the other half on the bottom strand, the unraveling is delayed since the slip-spring exerts a drag alternately first on one strand and then on the other, which frustrates chain unraveling. Interestingly, we previously showed that, starting from the kinked state, the strain at which EKD achieves full unraveling is almost twice that of affine motion as shown in [18] Figure 6, which matches with what find here with MEF. Thus, it seems that the slip-spring simulations under MEF assumption (a maximum force and re-location of slip spring’s anchoring points) follow the same chain evolution, both in stress and conformation, as in the EKD. This means that for the full coil-to-stretch evolution of the chain, one can use the slip spring simulations with MEF assumption. However, as mentioned before, the computational time of the slip spring simulations increases as the chains get longer, which may limit its use for comparison with experimental data.
